# A combination of cross-neutralizing antibodies synergizes to prevent SARS-CoV-2 and SARS-CoV pseudovirus infection

**DOI:** 10.1016/j.chom.2021.04.005

**Published:** 2021-05-12

**Authors:** Hejun Liu, Meng Yuan, Deli Huang, Sandhya Bangaru, Fangzhu Zhao, Chang-Chun D. Lee, Linghang Peng, Shawn Barman, Xueyong Zhu, David Nemazee, Dennis R. Burton, Marit J. van Gils, Rogier W. Sanders, Hans-Christian Kornau, S. Momsen Reincke, Harald Prüss, Jakob Kreye, Nicholas C. Wu, Andrew B. Ward, Ian A. Wilson

**Affiliations:** 1 Department of Integrative Structural and Computational Biology, The Scripps Research Institute, La Jolla, CA 92037, USA; 2 Department of Immunology and Microbiology, The Scripps Research Institute, La Jolla, CA 92037, USA; 3 Department of Medical Microbiology and Infection Prevention, Amsterdam University Medical Centers, Location AMC, University of Amsterdam, Amsterdam, the Netherlands; 4 Department of Microbiology and Immunology, Weill Medical College of Cornell University, New York, NY 10021, USA; 5 German Center for Neurodegenerative Diseases (DZNE) Berlin, Berlin, Germany; 6 Ragon Institute of MGH, MIT and Harvard, Cambridge, MA 02139, USA; 7 Neuroscience Research Center (NWFZ), Cluster NeuroCure, Charité-Universitätsmedizin Berlin, corporate member of Freie Universität Berlin, Humboldt-Universität Berlin, and Berlin Institute of Health, Berlin, Germany; 8 Helmholtz Innovation Lab BaoBab, Berlin, Germany; 9 Department of Neurology and Experimental Neurology, Charité-Universitätsmedizin Berlin, corporate member of Freie Universität Berlin, Humboldt-Universität Berlin, and Berlin Institute of Health, Berlin, Germany; 10 Department of Pediatric Neurology, Charité-Universitätsmedizin Berlin, corporate member of Freie Universität Berlin, Humboldt-Universität Berlin, and Berlin Institute of Health, Berlin, Germany; 11 Department of Biochemistry, University of Illinois at Urbana-Champaign, Urbana, IL 61801, USA; 12 Carl R. Woese Institute for Genomic Biology, University of Illinois at Urbana-Champaign, Urbana, IL 61801, USA; 13 Center for Biophysics and Quantitative Biology, University of Illinois at Urbana-Champaign, Urbana, IL 61801, USA; 14 The Skaggs Institute for Chemical Biology, The Scripps Research Institute, La Jolla, CA 92037, USA

**Keywords:** cross-neutralizing antibody, antibody cocktail, synergy, coronavirus, SARS-CoV-2, COVID-19, antibody-antigen interaction, 3D structure, crystallography

## Abstract

Coronaviruses have caused several human epidemics and pandemics including the ongoing coronavirus disease 2019 (COVID-19). Prophylactic vaccines and therapeutic antibodies have already shown striking effectiveness against COVID-19. Nevertheless, concerns remain about antigenic drift in SARS-CoV-2 as well as threats from other sarbecoviruses. Cross-neutralizing antibodies to SARS-related viruses provide opportunities to address such concerns. Here, we report on crystal structures of a cross-neutralizing antibody, CV38-142, in complex with the receptor-binding domains from SARS-CoV-2 and SARS-CoV. Recognition of the N343 glycosylation site and water-mediated interactions facilitate cross-reactivity of CV38-142 to SARS-related viruses, allowing the antibody to accommodate antigenic variation in these viruses. CV38-142 synergizes with other cross-neutralizing antibodies, notably COVA1-16, to enhance neutralization of SARS-CoV and SARS-CoV-2, including circulating variants of concern B.1.1.7 and B.1.351. Overall, this study provides valuable information for vaccine and therapeutic design to address current and future antigenic drift in SARS-CoV-2 and to protect against zoonotic SARS-related coronaviruses.

## Highlights

• X-ray and EM structures of CV38-142 with SARS-CoV-2 and SARS-CoV RBDs and spikes
• N343 glycan and bound waters facilitate cross-reactivity to SARS-related viruses
• CV38-142 synergizes with CR3022 conserved site antibodies, in particular COVA1-16
• These two cross-neutralizing antibodies neutralize SARS-CoV-2 variants of concern

## Graphical abstract

**Figure:**
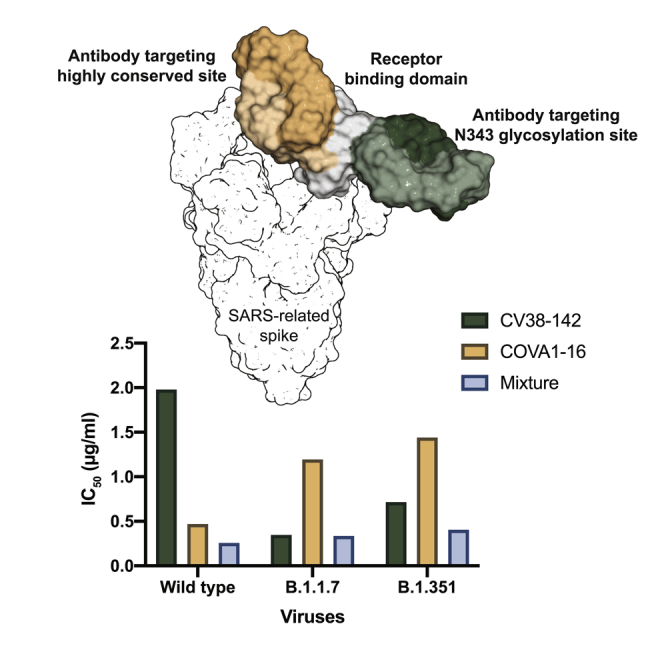

## Teaser

Antibody CV38-142 from a COVID-19 patient cross-reacts with SARS-related viruses. Liu et al. reveal that CV38-142 interacts with the S309 N343 glycan site and synergizes with COVA1-16 that binds the conserved CR3022 site. Combination of the two cross-neutralizing antibodies shows enhanced neutralization to circulating variants of concern B.1.1.7 and B.1.351.

## Introduction

Severe acute respiratory syndrome coronavirus (SARS-CoV), Middle East respiratory syndrome coronavirus (MERS-CoV), and SARS-CoV-2 have caused epidemics in the past two decades, including the current pandemic of coronavirus disease 2019 (COVID-19). SARS-CoV-2 has already resulted in more than 140 million reported cases and over 3 million deaths worldwide as of April 21, 2021 (https://covid19.who.int). Although these viruses have devastating consequences in the human population, they are of animal origin and have less morbidity or even no symptoms in their animal hosts (Cui et al., 2019; Tortorici and Veesler, 2019; Ye et al., 2020). In addition to these human β-coronaviruses (SARS-CoV, MERS-CoV, and SARS-CoV-2), other SARS-related coronaviruses (SARSr-CoVs) of the sarbecovirus subgenus within the β-coronavirus genus are found in mammalian reservoirs, such as bats and pangolins, and could also constitute potential pandemic threats to human health (Hu et al., 2015; Lam et al., 2020; Wacharapluesadee et al., 2021; Ye et al., 2020). Recently, mutations in SARS-CoV-2 were identified in farmed mink, and these viruses were found to be reciprocally transmissible between humans and farmed mink (Welkers et al., 2021), further underscoring concerns about the long-term efficacy of current antibody therapies and vaccines under development (Mallapaty, 2020). Hence, identification and characterization of cross-neutralizing antibodies within the sarbecovirus subgenus are of value for design and development of therapeutics and next generation vaccines to mitigate against antigenic drift as well as future SARSr-CoV transmission to humans from the mammalian reservoir.

Since the spike protein is the major surface protein on sarbecoviruses, neutralizing antibodies are targeted toward the spike, and many of these antibodies are able to prevent virus interaction with the host receptor, angiotensin-converting enzyme 2 (ACE2) (Piccoli et al., 2020; Yuan et al., 2021b). Other inhibition mechanisms also seem to be possible and are being assessed for other subsets of antibodies (Hansen et al., 2020; Piccoli et al., 2020; Pinto et al., 2020). The receptor-binding domain (RBD) of the spike protein is highly immunogenic and can induce highly specific and potent neutralizing antibodies (nAbs) against SARS-CoV-2 virus (Barnes et al., 2020a, 2020b; Brouwer et al., 2020; Cao et al., 2020; Ju et al., 2020; Kreye et al., 2020; Piccoli et al., 2020; Robbiani et al., 2020; Rogers et al., 2020; Yuan et al., 2020a; Zost et al., 2020). Many of these nAbs bind to the receptor-binding motif (RBM) on the RBD (Yuan et al., 2021b). However, the breadth of these nAbs is limited as the RBM shares relatively low sequence identity among sarbecoviruses; the RBM is only 48% conserved between SARS-CoV-2 and SARS-CoV compared to 73% for the complete RBD (84% identity for non-RBM regions of the RBD). The RBD is also prone to naturally occurring mutations, along with the N-terminal domain (NTD) and other non-RBD and non-NTD regions, where insertions and deletions have also been found (Greaney et al., 2021b; Kemp et al., 2021; Liu et al., 2021; McCarthy et al., 2021; Starr et al., 2020; Tegally et al., 2021; Van Egeren et al., 2020; Voloch et al., 2021). Recent studies showed that many potent monoclonal nAbs are sensitive to the antigenic drift or mutation on the RBD of the spike protein (Thomson et al., 2021; Wang et al., 2021a, 2021b; Weisblum et al., 2020; Wibmer et al., 2021), as well as polyclonal sera from convalescent or vaccinated individuals (Andreano et al., 2020; Greaney et al., 2021a; Liu et al., 2021; Wang et al., 2021a, 2021b; Weisblum et al., 2020; Wu et al., 2021).

We and others have reported cross-neutralizing antibodies such as COVA1-16, H014, EY6A, and S304 that bind to a highly conserved cryptic site in RBD of the spike (Liu et al., 2020; Lv et al., 2020; Piccoli et al., 2020; Yuan et al., 2021b; Zhou et al., 2020). Although the epitopes of these antibodies do not overlap with the ACE2 receptor-binding site, some can sterically block ACE2 binding to the RBD or attenuate ACE2 binding affinity (Liu et al., 2020; Lv et al., 2020). Other RBD surfaces are also possible targets for cross-neutralizing antibodies but are only moderately conserved within sarbecoviruses, although more so than the RBM. Such a site was originally identified as the epitope for antibody S309, which was isolated from a SARS patient, but cross-neutralizes SARS-CoV-2. S309 binds to a non-RBM surface containing an N-glycosylation site at N343 (Pinto et al., 2020). Further investigation is ongoing as to whether the S309 site is a common target for antibodies elicited by SARS-CoV-2 infection. Here, we report on cross-neutralization of sarbecoviruses by an *IGHV5-51*-encoded antibody isolated from a SARS-CoV-2 patient. High-resolution crystal structures of CV38-142 were determined in complex with both SARS-CoV RBD and SARS-CoV-2 RBD in combination with another cross-neutralizing antibody, COVA1-16. The structural information, along with binding and neutralization data, revealed that CV38-142 can be combined with cross-neutralizing antibodies to other epitopes to generate therapeutic cocktails to protect against SARS-CoV-2 variants, escape mutants, and future zoonotic coronavirus epidemics. The information may also inform next generation vaccine and therapeutic design (Barnes et al., 2020a).

## Results

### CV38-142 neutralizes SARS-CoV-2 and SARS-CoV pseudoviruses and binds SARSr-virus RBDs

**Figure 1:**
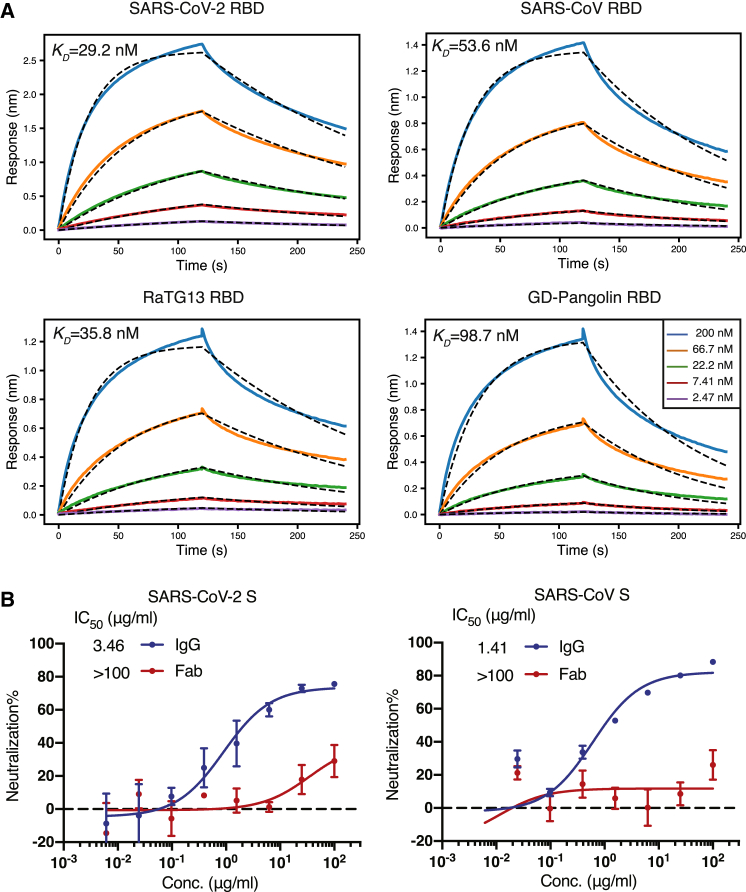
CV38-142 binds and cross-neutralizes SARS-CoV-2 and SARS-CoV (A) CV38-142 Fab binds to RBDs from human, bat, and pangolin sarbecoviruses with generally similar affinities. Binding kinetics were measured by biolayer interferometry (BLI) with RBDs on the biosensor and Fab in solution. Concentrations of Fab serial dilution are shown in the inset in the lower right panel. The association and disassociation were recorded in real time (s) on the x axis with binding response (nm) on the y axis with colored lines. Disassociation constant (K_D_) values were obtained by fitting a 1:1 binding model. The fitted curves are represented by the dashed lines (black). (B) CV38-142 neutralizes both SARS-CoV-2 and SARS-CoV, while its Fab counterpart barely neutralizes the two pseudotype viruses at the highest concentrations tested in the same neutralization assay. The IgG half-maximal inhibitory concentration (IC_50_) values (3.46 μg/mL for SARS-CoV-2 and 1.41 μg/mL for SARS-CoV) were determined using Prism software (version 8.4.3). Error bars indicate standard deviation (SD) of at least two biological replicates.

Previously, we reported that antibody CV38-142 isolated from a COVID-19 patient showed potent neutralization on authentic SARS-CoV-2 virus (Munich isolate 984) and was able to cross-react with SARS-CoV (Kreye et al., 2020). CV38-142 is an *IGHV5-51*-encoded antibody with little somatic hypermutation (only four mutations in the amino-acid sequence). This germline heavy-chain gene was also used in another cross-reactive antibody, CR3022 (Yuan et al., 2020b), that was isolated from a SARS patient (ter Meulen et al., 2006), but their CDRH3s are quite distinct. A biolayer interferometry (BLI) binding assay revealed that CV38-142 binds with high affinity not only to SARS-CoV-2 RBD (29 nM) but also SARS-CoV, RaTG13, and Guangdong pangolin coronavirus RBDs with roughly comparable affinity (36–99 nM) (Figure 1A). A pseudovirus neutralization assay showed that CV38-142 immunoglobulin G (IgG) neutralizes both SARS-CoV-2 and SARS-CoV with similar potency (3.5 and 1.4 μg/mL) (Figure 1B). The roughly comparable binding to RaTG13 and Guangdong pangolin coronavirus RBDs suggests that CV38-142 may also neutralize these zoonotic SARS-related viruses (SARSr viruses). Of note, the CV38-142 Fab exhibits much weaker or no neutralization in the same assay, which suggests that the avidity of bivalent CV38-142 IgG plays a crucial role in the neutralization (Figure 1B) as we also observed in other antibodies such as COVA1-16 (Liu et al., 2020).

### CV38-142 can be combined with either RBM or CR3022 cryptic site antibodies

**Figure 2:**
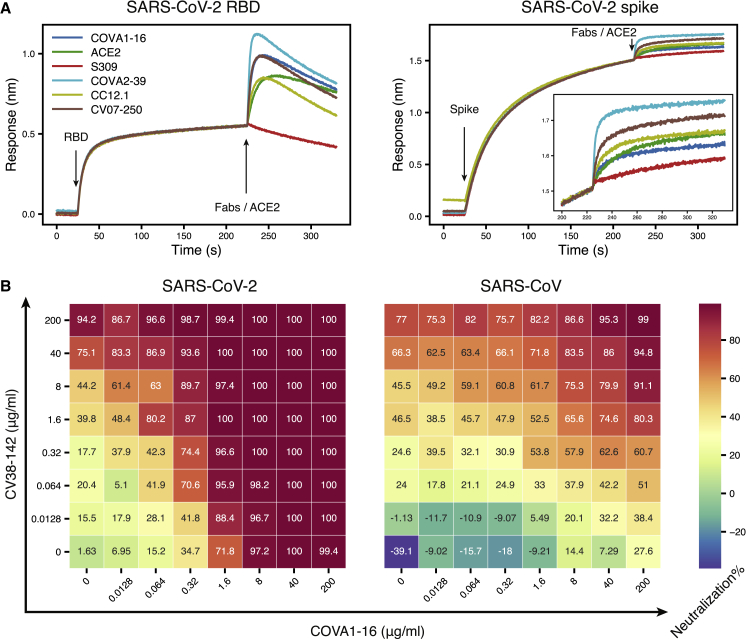
CV38-142 can be combined with antibodies to the receptor binding site or CR3022 cryptic site (A) Competitive binding of CV38-142 to SARS-CoV-2 RBD or spike. Inset in the right panel shows a zoomed-in view for Fabs/ACE2 binding on spike. A sandwich binding assay was used for the competition assay. CV38-142 IgG was first pre-loaded on the biosensor, then SARS-CoV-2 RBD or spike was loaded at the indicated time point. The biosensors with captured antibody-antigen complex were tested against binding to a second antibody Fab or human ACE2. Loading events for RBD/spike and the second antibody Fab/ACE2 are indicated by arrows along the timeline (x axis), while the binding response (nm, y axis) was recorded in real time as colored lines corresponding to each antibody Fab or ACE2. (B) Cross-neutralization dose-response matrix of an antibody cocktail consisting of CV38-142 and COVA1-16. The pseudovirus neutralization assay was performed by addition of mixtures of varying ratios of CV38-142 and COVA1-16. The percentage neutralization for each experiment with SARS-CoV-2 and SARS-CoV is plotted on heatmap matrices with their corresponding color bar shown on the right. See also Figure S1.

Recent reports on SARS-CoV-2 mutations in both human and mink populations give rise to concerns about viral escape from current vaccines and therapeutics in development (Andreano et al., 2020; Greaney et al., 2021a; Kemp et al., 2021; Mallapaty, 2020; Oude Munnink et al., 2021; Tegally et al., 2021; Voloch et al., 2021). However, antibody cocktails that bind to distinct epitopes can increase neutralization breadth and may help prevent escape mutations (Baum et al., 2020; Du et al., 2020; Greaney et al., 2021b; Hansen et al., 2020; Koenig et al., 2021). We previously reported that CV38-142 does not compete for RBD binding with other potent antibodies in our sample set, which are encoded by diverse germline genes, such as CV07-200 (*IGHV*1-2), CV07-209 (*IGHV*3-11), CV07-222 (*IGHV*1-2), CV07-250 (*IGHV*1-18), CV07-262 (*IGHV*1-2), CV38-113 (*IGHV*3-53), and CV38-183 (*IGHV*3-53) (Kreye et al., 2020). Here, we show that CV38-142 can bind either SARS-CoV-2 RBD or spike protein at the same time in a sandwich assay as CC12.1 and COVA2-39 (Figure 2A), which are potent *IGHV*3-53 nAbs from different cohorts (Brouwer et al., 2020; Rogers et al., 2020). Since CC12.1 (Yuan et al., 2020a), COVA2-39 (Wu et al., 2020), and CV07-250 (Kreye et al., 2020) bind to the RBM, these data suggest that CV38-142 can be combined with potent RBM antibodies derived from diverse germlines in an antibody cocktail. Hence, we tested whether CV38-142 could bind RBD at the same time as two other potent cross-neutralizing antibodies that target other sites on the RBD (Yuan et al., 2021b). The sandwich binding assay revealed that CV38-142 competes with S309 from a SARS patient (Pinto et al., 2020) but is compatible with COVA1-16, a cross-neutralizing antibody to the CR3022 site isolated from a COVID-19 patient (Figure 2A) (Brouwer et al., 2020). We then assembled a cocktail consisting of different amounts and ratios of CV38-142 and COVA1-16. The cocktail showed enhanced potency in the 2D neutralization matrix assay with SARS-CoV-2 and enhanced potency and improved efficacy with SARS-CoV pseudoviruses, demonstrating that CV38-142 is a promising candidate for pairing with cross-neutralizing antibodies to the highly conserved CR3022 cryptic site (Figure 2B). For example, 100% inhibition in the neutralization assay could be achieved with 1.6 μg/mL of each of CV138-142 and COVA1-16 with SARS-CoV-2 compared to >200 μg and 40 μg/mL for the individual antibodies, respectively. For SARS-CoV, the corresponding numbers were higher and required 200 μg/mL of each antibody to approach 100% inhibition, where 200 μg only achieved 77% and 28% neutralization, respectively, for each individual antibody. These changes in potency and efficacy suggest synergy between CV38-142 and COVA1-16. Synergistic neutralization effects have been analyzed in other viruses (Zwick et al., 2001), including coronaviruses (Pinto et al., 2020; ter Meulen et al., 2006; Zost et al., 2020), and can be quantified by several algorithms using multiple synergistic models (Ianevski et al., 2017; Wooten and Albert, 2020). Using the most up-to-date synergy model, our data analysis showed synergistic potency (α > 1) between CV38-142 and COVA1-16 in two directions against both SARS-CoV-2 and SARS-CoV pseudoviruses, which suggests reciprocal synergy between CV38-142 and COVA1-16 (Figure S1). Addition of COVA1-16 also improved the maximal efficacy of CV38-142 in neutralizing SARS-CoV as indicated by the positive synergistic efficacy score (β > 0) (Figure S1) as well as the neutralization matrix (Figure 2B).

### CV38-142 binds to a proteoglycan site on SARS-CoV-2 RBD

**Figure 3:**
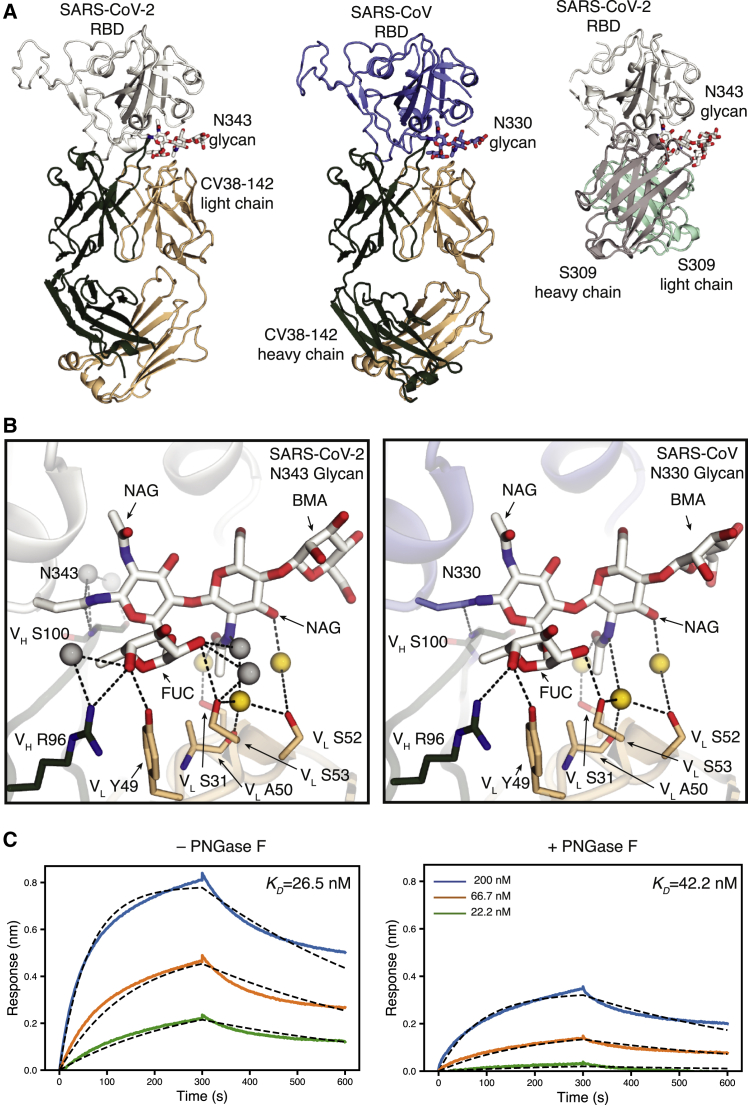
The CV38-142 epitope on the RBD involves an N-glycosylation site on SARS-CoV-2 and SARS-CoV (A) Ribbon representation of the crystal structures of SARS-CoV-2 (left) and SARS-CoV (middle) RBD in complex with CV38-142 Fab and comparison to cryo-EM structure of S309 Fab in complex with spike trimer (PDB: 6WPS) (right, only the comparable RBD regions are shown). CV38-142 Fab heavy chain is in forest green and light chain in wheat, S309 Fab heavy chain in gray and light chain in cyan, SARS-CoV-2 RBD in white, and SARS-CoV RBD in pale blue. The N343 glycan in SARS-CoV-2 and N330 glycan in SARS-CoV are shown as sticks. The same perspective views are used for the comparison. The overall structure of SARS-CoV-2 RBD in complex with CV38-142 and COVA1-16 is shown in Figure S1A. (B) Interactions between CV38-142 Fab residues and N343 (SARS-CoV-2) and N330 (SARS-CoV) glycans are shown in stick representation. Water molecules mediating the antibody-antigen interaction are shown in spheres (gray; yellow for shared water-mediated interactions between SARS-CoV-2 and SARS-CoV). Dashed lines (black) represent hydrogen bonds. Residues of the heavy and light chain are both involved in the interactions with glycans. The interactions of CV38-142 with SARS-CoV-2 RBD and SARS-CoV RBD are similar. (C) Glycan removal in the RBD decreases binding between CV38-142 and SARS-CoV-2 RBD. The binding kinetics were measured by BLI with CV38-142 Fab on the biosensor and RBD in solution. SARS-CoV-2 RBD was pretreated with or without PNGase F digestion in the same concentration and condition before being used in the BLI assay. Concentrations of RBD serial dilution are shown in the right panel. The association and disassociation were recorded in real time (s) in the x axis and response (nm) on the y axis as colored lines. Disassociation constant (K_D_) values were obtained by fitting a 1:1 binding model with fitted curves represented by the dash lines. See also Figure S2 and Table S1.

We then determined the crystal structure of SARS-CoV-2 RBD in complex with CV38-142 and COVA1-16 Fabs at 1.94 Å resolution (Figure 3A; Figure S2A; Table S1). COVA1-16 binds to a highly conserved epitope on RBD in the same approach angle as we reported before (Liu et al., 2020). However, CV38-142 binds to a less conserved surface with no overlap with the COVA1-16 epitope (Liu et al., 2020) and involves the N-glycosylation site at N343 on the RBD that is distal to the RBM (Figure 3A; Figure S2A). This N343 glycosylation site is conserved in sarbecoviruses (Figure S3). The crystal structure showed well-resolved density for four of the sugar moieties attached to N343 (Figure S4A). Several hydrogen bonds are made to the glycan from both heavy and light chain (Figure 3B). The V_H_ S100 amide hydrogen bonds to the post-translationally modified N343, and V_H_ R96, V_L_ Y49, and V_L_ S53 hydrogens bond to the core fucose moiety of the glycan as well as water molecules that mediate interactions between CV38-142 and glycan. These interactions contribute to binding between CV38-142 and SARS-CoV-2 RBD as glycan removal from the RBD using PNGase F, or with RBD expressed in HEK293S cells that results in high mannose glycans with no core fucose (Reeves et al., 2002), results in a decrease in binding to CV38-142 from a K_D_ (disassociation constant) of 27 nM to 42nM and 168 nM (Figure 3C; Figures S2B and S2C). Glycan removal resulted in only a slight decrease in binding to SARS-CoV-elicited antibody, S309 (Figure S2E), which also interacts with the N343 glycan in SARS-CoV-2 RBD (Pinto et al., 2020). To eliminate glycosylation at the N343 site, mutations were introduced into the NxT sequon either at asparagine or threonine residue in both SARS-CoV-2 and SARS-CoV RBDs. An enzyme-linked immunosorbent assay (ELISA) showed a significant drop in binding of CV38-142 to both SARS-CoV-2 and SARS-CoV RBD, while antibody binding to other epitopes, such as CR3022 and CV07-209, were not impacted (Figure S2D). Deep mutational scanning on SARS-CoV-2 RBD previously indicated lower expression of mutants with changes near the glycosylation site, especially at residue 343 (Starr et al., 2020). We therefore used S309 as a probe to show the epitope surface is exposed and can be recognized by S309 (Figure S2D). S309 is less affected by the absence of the N343 glycan as mutation in the NxT sequon at residue 345 had minor impact on S309 binding to the RBD, although there was a significant drop in binding to SARS-CoV-2 RBD N343Q (Figure S2D). Residue 343 also appears to be less tolerant of mutations than residue 345 (Starr et al., 2020). These findings suggest that the complex glycan at N343 (Wang et al., 2020; Watanabe et al., 2020) contributes to RBD binding by CV38-142, especially with its core fucose, rather than simply acting as a glycan shield to antibodies.

**Figure 4:**
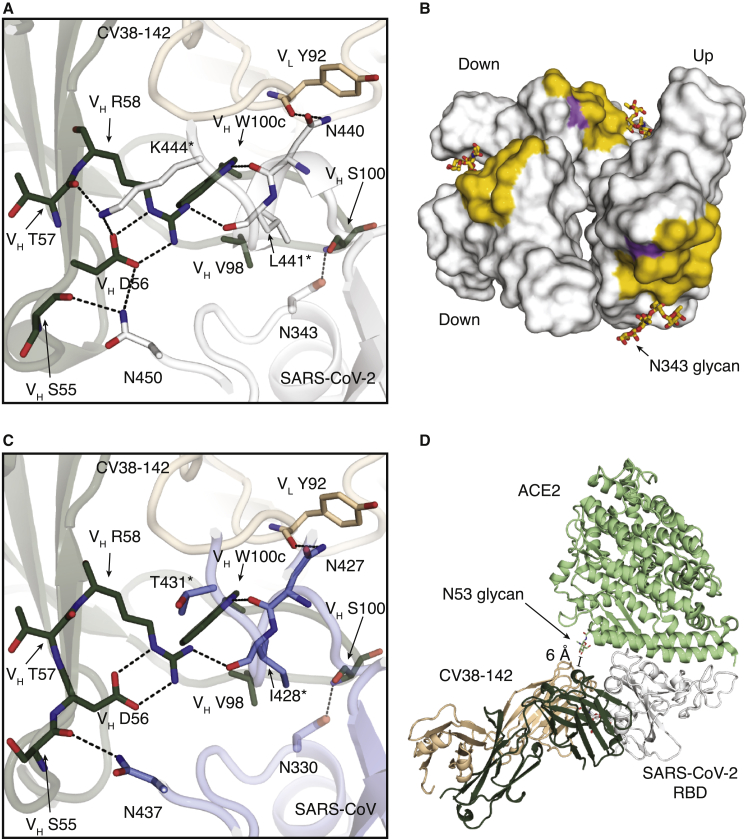
Molecular interactions between CV38-142 and RBDs SARS-CoV-2 RBD is in white, SARS-CoV RBD in pale blue, CV38-142 heavy chain in forest green and light chain in wheat, and ACE2 in pale green. Corresponding residues that differ between SARS-CoV-2 and SARS-CoV are labeled with asterisks (^∗^). Dashed lines (black) represent hydrogen bonds or salt bridges. (A) Direct interactions between CV38-142 and SARS-CoV-2 RBD are shown in sticks. (B) Surface representation of the CV38-142 epitope site in SARS-CoV-2 RBD. The CV38-142 epitope is exposed to solvent regardless of whether the RBD is in the “up” or “down” state. RBDs are shown in surface representation model with symmetry derived from the spike protein (PDB: 6VYB) to show their solvent-accessible surface area in either “up” or “down” state. The buried surface area (BSA) was calculated by PISA program (Krissinel and Henrick, 2007). The epitope surface buried by the CV38-142 heavy chain is shown in orange and that by the light chain in purple. The total surface area buried on the RBD by CV38-142 is 792 Å^2^ with 629 Å^2^ (79%) contributed by the heavy chain and 163 Å^2^ (21%) by the light chain. (C) Direct interactions between CV38-142 and SARS-CoV RBD. The same perspective is used as in (A). (D) Structural alignment illustrating a model with simultaneous binding by CV38-142 and ACE2 to SARS-CoV-2 RBD. Structures of CV38-142 Fab + SARS-CoV-2 RBD and ACE2 + SARS-CoV-2 spike are aligned by superimposition of their RBD. The scale bar shows the closest distance between ACE2 and CV38-142, which is 6 Å, although some sugars in the N53 glycan are not visible in the electron density map. See also Figures S3 and S5 and Table S2.

In addition to the N343 glycan, interactions with other residues are observed between CV38-142 and SARS-CoV-2 RBD. The V_H_ R58 guanidinium hydrogen bonds to the L441 backbone carbonyl in SARS-CoV-2, while its hydrophobic portion interacts with the alkene region of K444. V_H_ W100c indole hydrogen bonds with the N440 carbonyl and forms a hydrophobic patch with V_H_ V98 and the L441 side chain in SARS-CoV-2 (Figure 4A). The V_H_ S55 backbone carbonyl oxygen hydrogen bonds to the N450 amide (Figure 4A). Besides heavy-chain interactions, the V_L_ Y92 carbonyl oxygen hydrogen bonds to the N440 side chain in SARS-CoV-2 RBD. Overall, CV38-142 interacts with RBD mainly through its heavy chain, which contributes 79% of the buried surface area (BSA) on the RBD (629 Å^2^ out of 792 Å^2^ total BSA as calculated by the PISA program; Figure 4B). Eight polar interactions and two sites of hydrophobic interactions are involved in binding of CV38-142 to SARS-CoV-2 RBD (Table S2).

### CV38-142 uses a plethora of water-mediated interactions to aid cross-reactivity with SARS-CoV-2 and SARS-CoV

**Figure 5:**
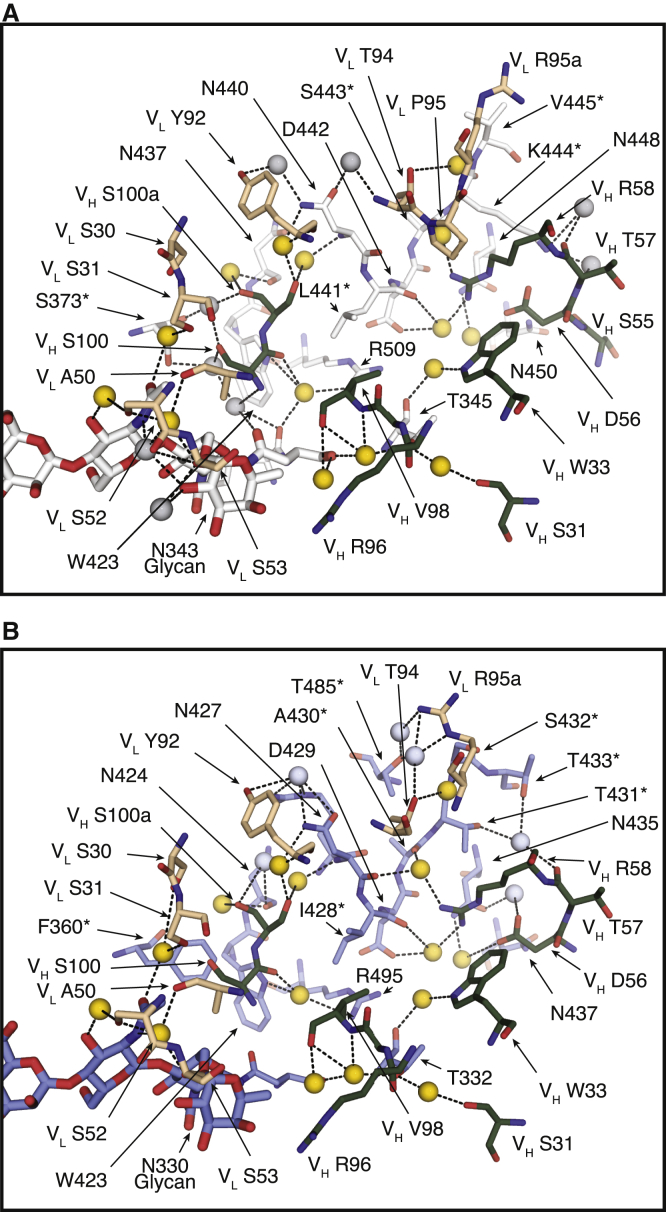
A plethora of water molecules mediate interactions between CV38-142 and SARS-CoV-2 and SARS-CoV RBD SARS-CoV-2 RBD is in white, SARS-CoV RBD in pale blue, CV38-142 heavy chain in forest green and light chain in wheat. Corresponding residues that differ between SARS-CoV-2 and SARS-CoV are labeled with asterisks (^∗^). Dashed lines (black) represent hydrogen bonds. Amino acid residues as well as the glycans involved in the water-mediated interactions are shown in sticks. Yellow spheres indicate water molecules in the same location in the structures of the CV38-142 Fab + SARS-CoV-2 RBD + COVA1-16 Fab complex (A) and the CV38-142 Fab + SARS-CoV RBD (B). Grey spheres indicate unique water molecules in each complex structure. See also Figure S4.

The RBD residues involved in CV38-142 interaction with SARS-CoV-2 are not all identical in SARS-CoV RBD (Figure S3). Ten of 20 residues differ in the CV38-142 epitope between SARS-CoV-2 and SARS-CoV. To investigate how CV38-142 accommodates these differences, we determined a crystal structure of CV38-142 Fab in complex with SARS-CoV RBD at 1.53 Å resolution (Figure 3A; Table S1). CV38-142 binds SARS-CoV RBD at the same site with an identical approach angle, albeit interacting with some different residues in the RBD. Interaction with the conserved N330 glycan (Figure 3B; Figure S2D) and the conserved N427 and N437 (Figure 4C) are the same as with SARS-CoV-2. Similar hydrophobic interactions are maintained with I428 in SARS-CoV RBD and L441 in SARS-CoV-2 RBD (Figure 4C). However, interactions with K444 are lost in CV38-142 binding to SARS-CoV RBD due a change to the corresponding T431 in SARS-CoV RBD (Table S2). A hydrophilic surface of CDRH3 of CV38-142 is now juxtaposed to F360 of SARS-CoV RBD compared to S373 of SARS-CoV-2 RBD. The phenyl moiety of F360 adopts heterogeneous conformations with diffuse electron density in the X-ray structure (Figure S4E). Side chains of other epitope residues of SARS-CoV RBD that differ from SARS-CoV-2 RBD are well adapted to the binding interface with no clashes or significant changes in the CV38-142 structure. Thus, the overall binding of CV38-142 to SARS-CoV RBD is essentially identical to SARS-CoV-2 (Figures 1A and 3A) despite a few differences in specific interactions (Figures 4A and 4C). It would appear to be unusual that the binding between an antibody and antigen would be retained at the same level with half of the polar interactions being depleted in the interface of a cross-reacting protein (Table S2). One explanation is the abundance of water molecules mediating interaction between CV38-142 and both SARS-CoV-2 and SARS-CoV RBD. Many conserved water-mediated interactions are found with the peptide backbone in both SARS-CoV-2 and SARS-CoV RBD (Figure 5). The structures here are at high enough resolution to confidently identify these bound water molecules (Figures S4C and S4D). Water molecules have also been shown to be particularly important in other antibody-antigen interfaces (Braden et al., 1995; Wilson and Stanfield, 1993; Yokota et al., 2003). The shape complementarity (SC) (Lawrence and Colman, 1993) between CV38-142 and SARS-CoV-2 or SARS-CoV (0.63 and 0.58, respectively) is lower than for the average for antibody-antigen interactions or protein-protein interactions (Kuroda and Gray, 2016), when water molecules are not considered. Consistent with the SC analysis and high binding affinities, 24 water molecules mediate more than 60 hydrogen bonds between CV38-142 and SARS-CoV-2 RBD (Figure 5A; Figure S4C). A comparable number of water-mediated interactions are also observed with SARS-CoV RBD (Figure 5B; Figure S4D). These water-mediated interactions are mostly conserved in the interaction with CV38-142 with SARS-CoV-2 and SARS-CoV RBDs, with 15 that overlap and mediate interactions with both SARS-CoV-2 and SARS-CoV RBD (Figure 5). Considering the contribution from these water molecules, the loss of some direct contacts between CV38-142 and SARS-CoV RBD may be partially compensated by these abundant water-mediated interactions, suggesting a potential mechanism whereby CV38-142 could resist antigenic drift.

### CV38-142 accommodates rather than competes with ACE2 binding to the RBD

Structure superimposition of ACE2 bound to RBD reveals no clash between ACE2 and CV38-142. The closest distance is 6 Å, which corresponds to the distance between the first NAG moiety of the ACE2 N53 glycan and the H66 imidazole of CV38-142 in the antibody complex with SARS-CoV-2 RBD (Figure 4D). There seems to be sufficient space for the remainder of the glycan to be accommodated due to the large open void between CV38-142 and ACE2. In addition, we observe some flexibility in this region (S60–H66) of CV38-142 that would allow even more room for the ACE2 N53 glycan if both ACE2 and CV38-142 were to bind RBD simultaneously (Figure S5A). BLI sandwich binding assays and the surface plasma resonance (SPR) competition assays revealed that binding of CV38-142 IgG does not occlude ACE2 binding to SARS-CoV-2 RBD or spike protein (Figure 2A; Figure S5B), suggesting no steric block between CV38-142 and ACE2. Since CV38-142 IgG potently neutralizes infection by SARS-CoV-2 and SARS-CoV pseudoviruses (Figure 1B) and in authentic virus assays (Kreye et al., 2020), this finding then poses a question about the mechanism of CV38-142 neutralization of sarbecovirus infection. One explanation is that CV38-142 somehow attenuates ACE2 or other cofactor binding that cannot be observed in the sandwich binding assay or the SPR competition assay. We in fact previously reported that CV38-142 IgG reduced ACE2 binding to SARS-CoV-2 RBD by 27% in an ELISA (Kreye et al., 2020). The possible constraint on accommodating the N53 glycan in ACE2 upon simultaneous binding by CV38-142 IgG may contribute to this reduction on ACE2 binding in the ELISA (Kreye et al., 2020).

### CV38-142 binds RBD in either “up” or “down” state and could cross-link spikes

**Figure 6:**
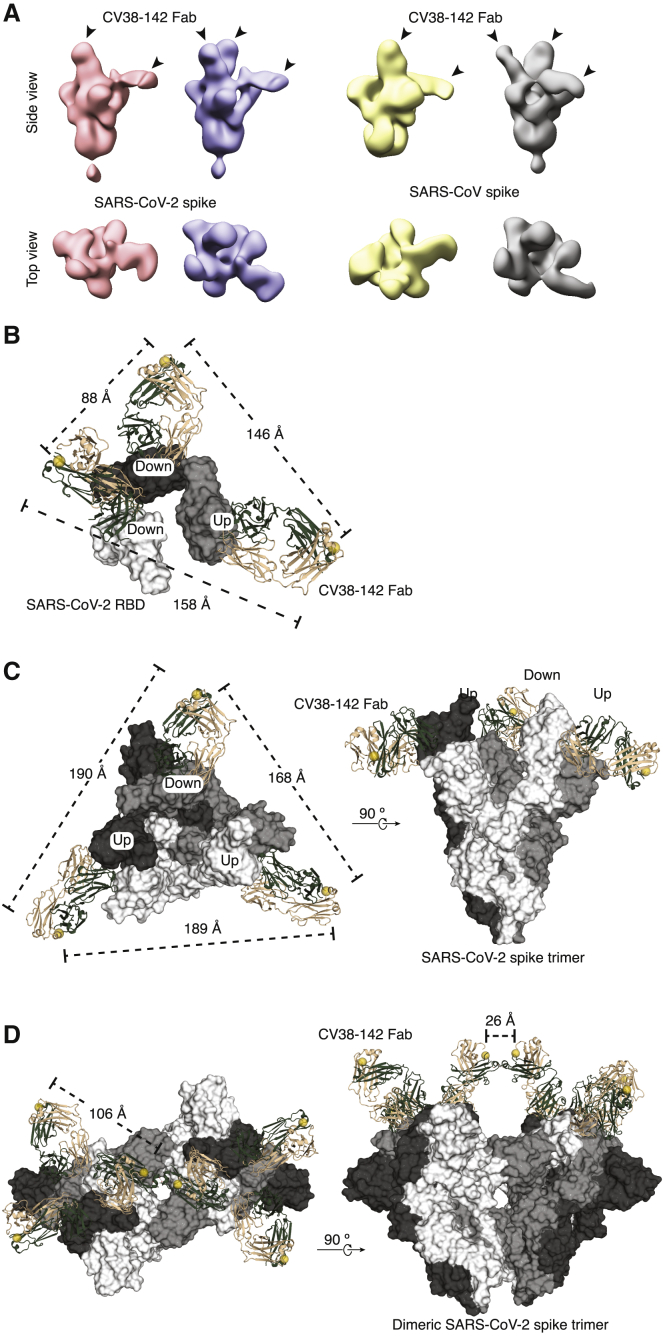
CV38-142 Fab binding to SARS-CoV-2 and SARS-CoV spike trimers by nsEM (A) CV38-142 Fab binding to spike trimers as observed by nsEM. Representative 3D nsEM reconstructions are shown of CV38-142 Fab complexed with the spike trimers with its RBDs in “up” and “down” states. The location of the bound CV38-142 Fabs are indicated by arrow heads. SARS-CoV-2 (pink) or SARS-CoV (yellow) spikes with at least one “up” RBD and one “down” RBD are bound by two CV38-142 Fabs. The spikes (pale blue to SARS-CoV-2 and gray to SARS-CoV) with RBD in the two “down,” one “up” states are bound by three Fabs. Other binding stoichiometries and conformations are show in Figure S6. (B–D) C-terminal distances of CV38-142 Fab binding to spikes. The three RBDs (B) or three protomers (C and D) in the spike trimer are shown in white, gray, and dark gray, respectively. CV38-142 Fabs are shown in ribbon representation with heavy chain in forest green and light chain in wheat. The C termini of CV38-142 heavy chains are shown as spheres (yellow). Dashed lines represent distances among the various combinations of C-termini. (B) nsEM fitting model. To measure the distances between C-termini of CV38-142 Fabs in nsEM data, the crystal structure of CV38-142 Fab + SARS-CoV-2 was fitted into the nsEM density in (A) (second from the left). (C and D) Structural superimposition of CV38-142 Fabs onto the spike trimer, which is shown in surface representation. Alignment of CV38-142 Fab binding to the spike trimer with RBD in two “up,” one “down” state (PDB: 7CAI) (C) or to a dimeric spike trimer that is found in Novavax vaccine candidate NVAX-CoV2373 with RBD in “all-down” state (PDB: 7JJJ) (Bangaru et al., 2020) (D). The (B–D) models represent various possibilities of CV38-142 binding to the spike protein on the viral surface. See also Figure S6.

Superimposition of the CV38-142 binding epitope onto a cryogenic electron microscopy (cryo-EM) structure of the spike trimer (PDB: 6VYB) suggests that CV38-142 is capable of binding RBD in both “up” and “down” states (Figure 4B). Consistent with this notion, 2D classification of the negative-stain electron microscopy (nsEM) images reveals that CV38-142 Fab can bind to SARS-CoV-2 or SARS-CoV spikes with various binding stoichiometries (Figure S6A and S6B). The 3D reconstructions of both SARS-CoV-2 and SARS-CoV spikes indicated that CV38-142 Fab could bind RBDs in either “up” or “down” state (Figure 6A; Figures S6C and S6D). The nsEM reconstructions also showed high flexibility of the RBD that only allowed reconstruction of partial density for the Fab (Figure S6D), suggesting heterogeneous conformations/dispositions of the RBD when bound with CV38-142 Fab. Since the resolutions of the nsEM data are insufficient to build atomic models of spikes, we fit the crystal structure of CV38-142 Fab + SARS-CoV-2 RBD into the nsEM density map of SARS-CoV-2 spike bound to three CV38-142 Fabs in the two “down,” one “up” state (Figure 6A, pale blue). The tentative fitting model suggests a distance of 88 Å between the heavy chain C termini of CV38-142 Fabs bound with RBD in “down” state and distances of 146 and 158 Å between the heavy-chain C terminus of CV38-142 Fab bound with RBD in “up” state and one of the RBDs in “down” state (Figure 6B).

For spike with RBDs in “two-up-one-down” state, we aligned CV38-142 Fab to the cryo-EM structure of SARS-CoV-2 spike (PDB: 7CAI) (Lv et al., 2020). Structural alignment suggests that the C terminus of the CV38-142 Fab heavy chain points away from the spike center axis due to its particular approach angle (Figure 6C), echoing a similar observation in the nsEM reconstruction data. The distance among the C termini ranges from 168–190 Å depending on the various combination of RBD states and is similar to that measured in the nsEM fitting model (Figure 6B), indicating that it is not possible for a CV38-142 IgG to bind two RBDs bivalently in either two “up” or one “up,” one “down” states within a spike trimer. For spike with RBDs in all “down” state, we aligned the crystal structure of CV38-142 Fab to a cryo-EM structure of dimeric spike trimer (PDB: 7JJJ). The structural alignment reveals that the distance between any two C termini of CV38-142 Fab bound within a spike trimer is around 106 Å, which also suggests that CV38-142 is unlikely to bind two RBDs in the “down” state within a spike trimer (Figure 6D). On the other hand, CV38-142 Fabs can bind RBDs from two adjacent spikes in a dimer seen in Novavax vaccine candidate NVAX-CoV2373 (Bangaru et al., 2020) with a distance of 26 Å between the C termini of the Fabs, suggesting that a CV38-142 IgG can bind a dimeric spike, or two spikes that are close together, with its two Fabs bound to RBDs from neighboring spikes (Figure 6D). These analyses are in line with the neutralization data, where bivalency plays a critical role on neutralizing both SARS-CoV-2 and SARS-CoV infection as the Fab has much weaker to no inhibition against pseudovirus infection by these sarbecoviruses (Figure 1B).

### A combination of CV38-142 and COVA1-16 showed enhanced potency on neutralizing SARS-CoV-2 variants

**Figure 7:**
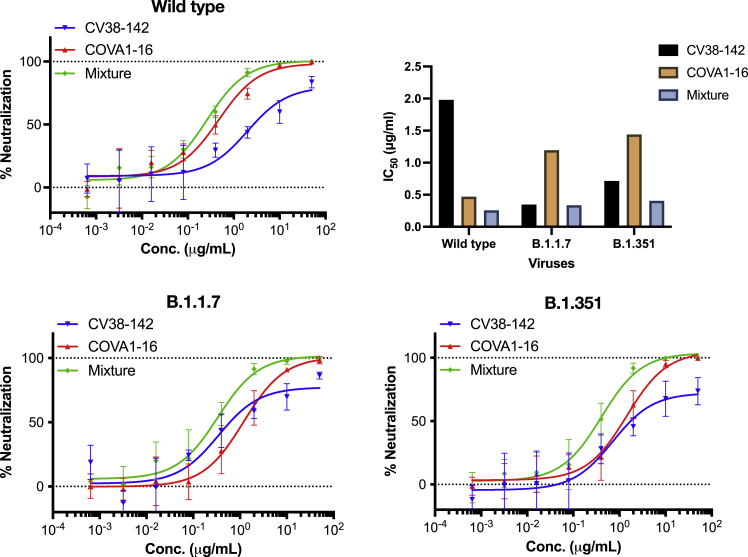
A combination of CV38-142 and COVA1-16 neutralizes circulating SARS-CoV-2 variants of concern Individual antibodies CV38-142, COVA1-16, and a mixture in a 1:1 molar ratio, were tested in a pseudovirus assay. CV38-142 showed similar potency (upper right panel) on neutralizing wild-type (Wuhan-Hu-1) SARS-CoV-2 pseudovirus (upper left panel) and two circulating variants of concern, i.e., B.1.1.7 isolated in the UK (lower left panel) and B.1.351 isolated in South Africa, namely 501Y.V2 (lower right panel). Although COVA1-16 showed a slight decrease in neutralization potency against B.1.1.7 and B.1.351, the combinatorial use of the two antibodies showed enhanced neutralization against all three viruses (upper right panel). Error bars indicate standard deviation (SD) of at least two biological replicates.

To examine whether the combination of two cross-neutralizing antibodies has superior neutralization to individual antibodies, we tested a mixture of the two with a 1:1 molar ratio against the SARS-CoV-2 wild-type (Wuhan-Hu-1) virus and two circulating variants of concern, i.e., B.1.1.7 and B.1.351, which escape from neutralization by many potent mAbs including some in Emergency Use Authorization and by convalescent plasma from some patients (Chen et al., 2021; Hoffmann et al., 2021; Wang et al., 2021a; Wibmer et al., 2021; Yuan et al., 2021a; Zhou et al., 2021). The neutralization data showed that CV38-142 neutralizes both B.1.1.7 and B.1.351 variants with enhanced potency, while COVA1-16 shows decreased potency. However, CV38-142 displays notable incomplete neutralization for all of these viruses (Figure 7), suggesting heterogeneity in the epitope recognized by the antibody. Consistent with the synergy analysis, the combination of the two antibodies showed enhanced potency (i.e., half-maximal neutralization concentration) as well as superior neutralization efficacy (i.e., maximum percentage of neutralization) compared to the individual antibodies alone in all three pseudovirus neutralization assays (Figure 7). The neutralization of pseudotyped SARS-CoV-2 virus with CV38-142 and COVA1-16 observed here is in line with prior neutralization of authentic SARS-CoV-2 (Brouwer et al., 2020; Kreye et al., 2020; Liu et al., 2020).

## Discussion

We report here on a distinct cross-neutralizing epitope in the RBD for an anti-SARS-CoV-2 neutralizing antibody, CV38-142, that cross-reacts with other sarbecoviruses including SARS-CoV-2, SARS-CoV, and SARSr viruses in pangolins and bats (Figure 1A; Figure S3). The epitope of CV38-142 is exposed to solvent regardless of whether RBD in the spike is in either the “up” or “down” states (Figure 4B). A SARS-CoV cross-neutralizing antibody S309, which has been previously characterized, binds to a nearby site and also interacts with the N343 glycan (Pinto et al., 2020). Both CV38-142 and S309 bind to the same face of the RBD to partially overlapping epitopes (Figure 3A; Figure S3) and compete with each other for RBD binding (Figure 2A). However, CV38-142 uses a different approach angle with its heavy and light chain rotated 90° around the epitope and N343 glycan site (N330 in SARS-CoV) compared to S309 (Figure 3A).

Binding of CV38-142 to the S309 epitope on the RBD allows simultaneous binding of RBM antibodies including those encoded by *IGHV3-53* and other germlines (Kreye et al., 2020) as well as others tested in this study. Moreover, we also found that a particular combination of cross-neutralizing antibodies, namely CV38-142 and COVA1-16, to two different sites could synergize to enhance neutralization of both SARS-CoV-2 and SARS-CoV pseudoviruses. The crystal structure of the antibody cocktail in complex with SARS-CoV-2 revealed how two different cross-neutralizing antibodies can interact with the RBD without inhibiting each other (Figure S2). Our neutralization data indicated enhanced potency (i.e., half-maximal inhibitory concentration) and efficacy (maximum percentage of inhibition) with the cross-neutralizing antibody combination (Figure 2B; Figure S1). The improved neutralization may arise from a synergistic effect on trapping the RBD in the “up” state since binding of COVA1-16 leads the RBD to tilt and twist in the “up” state (Liu et al., 2020). Since COVA1-16 is representative of cross-neutralizing antibodies that bind to the CR3022 cryptic site (Yuan et al., 2021b), other cross-neutralizing antibodies identified so far (i.e., S304, H014, and EY6A) (Lv et al., 2020; Piccoli et al., 2020; Zhou et al., 2020) that also bind to the CR3022 site (Figure S3) could also be paired with CV38-142 to improve cross-neutralization potency. The receptor binding site is quite diverse in sequence among SARS-CoV-2, SARS-CoV, and other SARSr viruses and has already been subject to escape mutations; thus, antibodies to cross-neutralizing sites may provide better protection against antigenic drift, and therefore we did not focus on combinations of antibodies that include those targeting the RBM due to their sensitivity to RBM mutations in the recent variants of concern. Furthermore, although CV38-142 binds to a less-conserved surface of the RBD across sarbecoviruses than COVA1-16, it uses fewer direct contacts and compensates through abundant water-mediated interactions that could accommodate antigenic differences and drift in sarbecoviruses. CV38-142 and COVA1-16 are capable of neutralizing the two main current variants of concern, i.e., B.1.1.7 and B.1.351. Combination of the two antibodies showed enhanced potency and efficacy in neutralizing SARS-CoV-2 as well as these variants of concern, thereby informing vaccine and antibody design and therapeutic use of such cross-neutralizing antibodies. Hence, our study provides valuable information to counteract potential escape mutations or antigenic drift in SARS-CoV-2, as well as future zoonotic viruses that could threaten global human health.

## STAR★Methods

### Key resources table

**Table:** 

REAGENT or RESOURCE	SOURCE	IDENTIFIER
ExpiCHO Expression System Kit	Thermo Fisher Scientific	Cat# A29133
Expi293 Expression System Kit	Thermo Fisher Scientific	Cat# A14635
Insect-XPRESS protein-free insect cell medium	Lonza Bioscience	Cat# 12-730Q
FreeStyle 293 expression medium	GIBCO	Cat# 12338002
Opti-MEM I reduced serum media	GIBCO	Cat# 51985091
Phosphate-buffered saline (PBS)	Thermo Fisher Scientific	Cat# 14040133
Ni-NTA Superflow	QIAGEN	Cat# 30450
Ni Sepharose excel	Cytiva	Cat# 17371202
DH10Bac competent cells	Thermo Fisher Scientific	Cat# 10361012
CaptureSelect CH1-XL Affinity Matrix	Thermo Fisher Scientific	Cat# 2943452010
Protein A column	Thermo Fisher Scientific	Cat# 17040301
Fetal Bovine Serum	Omega Scientific	Cat# FB-02

**Antibodies**		

Donkey anti-human IgG-HRPO	Dianova	Cat# 709-035-149, RRID: AB_2340495
Donkey F(ab’)2 anti-rabbit IgG-HRPO	Dianova	Cat# 711-036-152; RRID: AB_2340590

**Chemicals and Recombinant Proteins**		

DpnI	New England Biolabs	Cat# R0176L
Trypsin	New England Biolabs	Cat# P8101S
Fugene 6 Transfection Regent	Promega	Cat# E2691
Sodium chloride (NaCl)	Sigma-Aldrich	Cat# S9888
Tris Base	Sigma-Aldrich	Cat# 11814273001
Concentrated hydrochloric acid (HCl)	Sigma-Aldrich	Cat# H1758
Sodium azide (NaN_3_)	Sigma-Aldrich	Cat# S2002
Bovine Serum Albumin (BSA)	Sigma-Aldrich	Cat# A9418
Tween 20	Fisher Scientific	Cat# BP337-500
PEImax	Polysciences	Cat# 24765-1
Chemicals for protein crystallization	Hampton Research	N/A
1-step Ultra TMB-ELISA	Thermo Fisher Scientific	Cat# 34028

**Critical Commercial Assays**		

In-Fusion HD Cloning Kit	Takara	Cat# 639647
KOD Hot Start DNA Polymerase	EMD Millipore	Cat# 71086-3
PCR Clean-Up and Gel Extraction Kit	Clontech Laboratories	Cat# 740609.250
QIAprep Spin Miniprep Kit	QIAGEN	Cat# 27106
NucleoBond Xtra Maxi	Clontech Laboratories	Cat# 740414.100

**Deposited Data**		

X-ray coordinates and structure factors of CV38-142 Fab in complex with SARS-CoV-2 RBD and COVA1-16 Fab	This study	PDB: 7LM8
X-ray coordinates and structure factors of CV38-142 Fab in complex with SARS-CoV RBD	This study	PDB: 7LM9
Electron microscopy map of SARS-CoV spike in complex with Fab CV38-142 (three Fabs bound)	This study	EMDB: EMD-23469
Electron microscopy map of SARS-CoV spike in complex with Fab CV38-142 (two Fabs bound)	This study	EMDB: EMD-23470
Electron microscopy map of SARS-CoV-2 spike in complex with Fab CV38-142 (three Fabs bound)	This study	EMDB: EMD-23471
Electron microscopy map of SARS-CoV-2 spike in complex with Fab CV38-142 (two Fabs bound)	This study	EMDB: EMD-23472

**Cell Lines**		

ExpiCHO cells	Thermo Fisher Scientific	Cat# A29127; RRID: CVCL_5J31
Expi293F cells	Thermo Fisher Scientific	Cat# A14527; RRID: CVCL_D615
FreeStyle 293-F cells	Thermo Fisher Scientific	Cat# R79007; RRID: CVCL_D603
Expi293S cells	Thermo Fisher Scientific	Cat# A39240
HEK293T cells	ATCC	Cat# CRL-3216; RRID: CVCL_0063
Sf9 cells	ATCC	Cat# CRL-1711; RRID: CVCL_0549
High Five cells	Thermo Fisher Scientific	Cat# B85502; RRID: CVCL_C190
DH10Bac competent cells	Thermo Fisher Scientific	Cat# 10361012

**Recombinant DNA**		

phCMV3-COVA1-16 IgG heavy chain	(Brouwer et al., 2020)	N/A
phCMV3-COVA1-16 Fab heavy chain	(Brouwer et al., 2020)	N/A
phCMV3-COVA1-16 light chain	(Brouwer et al., 2020)	N/A
phCMV3-CV38-142 IgG heavy chain	This study	N/A
phCMV3-CV38-142 Fab heavy chain	This study	N/A
phCMV3-CV38-142 light chain	This study	N/A
pFastBac-SARS-CoV-RBD	(Yuan et al., 2020a)	N/A
pFastBac-SARS-CoV-2-RBD	(Yuan et al., 2020a)	N/A
phCMV3-ACE2	This study	N/A

**Software and Algorithms**		

HKL2000	(Otwinowski and Minor, 1997)	N/A
Phaser	(McCoy et al., 2007)	N/A
Coot	(Emsley et al., 2010)	N/A
Phenix	(Adams et al., 2010)	N/A
MolProbity	(Chen et al., 2010)	N/A
Sc	(Lawrence and Colman, 1993)	N/A
PyMOL	Schrödinger, LLC	RRID: SCR_000305
Appion	(Lander et al., 2009)	N/A
DoG Picker	(Voss et al., 2009)	N/A
Relion	(Scheres, 2012)	N/A
UCSF Chimera	(Pettersen et al., 2004)	N/A
Octet Analysis Software 12.0	Sartorius	https://www.moleculardevices.com

**Other**		

Fab-CH1 2nd generation (FAB2G) biosensors	Sartorius	Cat# 18-5125
Anti-Human Fc Capture (AHC) Biosensors	Sartorius	Cat# 18-5060
Ni-NTA biosensors	Sartorius	Cat# 18-5101
Streptavidin (SA) biosensors	Sartorius	Cat# 18-5057
Biotin CAPture Kit, Series S	Cytiva	Cat# 28920234
HiLoad 16/600 Superdex 200 pg	Cytiva	Cat# 28989335
HiLoad 16/600 Superose 6 pg	Cytiva	Cat# 29323952
Negative stain EM grids, 400 mesh	Electron Microscopy Sciences	Cat# EMS400-CU

### Resource availability

#### Lead contact

Further information and requests for resources and reagents should be directed to and will be fulfilled by the Lead Contact, Ian A. Wilson (wilson@scripps.edu).

#### Materials availability

All reagents generated in this study are available from the Lead Contact (I.A.W.) with a completed Materials Transfer Agreement.

#### Data and code availability

X-ray coordinates and structure factors have been deposited in the RCSB Protein Data Bank (PDB). The EM maps have been deposited at the Electron Microscopy Data Bank (EMDB). The accession number for CV38-142 Fab in complex with SARS-CoV-2 RBD and COVA1-16 Fab reported in this paper is PDB: 7LM8. The accession number for CV38-142 Fab in complex with SARS-CoV RBD reported in this paper is PDB: 7LM9. The accession number for SARS-CoV spike in complex with Fab CV38-142 (three Fabs bound) reported in this paper is EMDB: EMD-23469. The accession number for SARS-CoV spike in complex with Fab CV38-142 (two Fabs bound) reported in this paper is EMDB: EMD-23470. The accession number for SARS-CoV-2 spike in complex with Fab CV38-142 (three Fabs bound) reported in this paper is EMDB: EMD-23471. The accession number for SARS-CoV-2 spike in complex with Fab CV38-142 (two Fabs bound) reported in this paper is EMDB: EMD-23472. CV38-142 IGVH and IGVK sequences are available in GenBank: MW002785 and MW002803. COVA1-16 IGVH and IGVK sequences are available in GenBank: MT599835 and MT599919.

### Experimental model and subject details

#### Cell lines

ExpiCHO cells (Thermo Fisher Scientific Cat# A29127, RRID: CVCL_5J31) were cultured in ExpiCHO expression medium according to the manufacturer’s instructions and were used to express antibody IgGs and Fabs used in this study. DH10Bac competent cells (Thermo Fisher Scientific Cat# 10361012) were cultured in LB medium and used to generate bacmids containing the SARSr RBD coding sequences. Sf9 (Cat# CRL-1711, RRID: CVCL_0549) and High five cells (Thermo Fisher Scientific Cat# B85502, RRID: CVCL_C190) were cultured in Insect-XPRESS protein-free insect cell medium (Lonza Bioscience Cat# 12-730Q) according to the manufacturer’s instructions and used for generating baculoviruses and for expression of SARSr RBDs for crystallization and binding assays. Expi293F (Thermo Fisher Scientific Cat# A14527, RRID: CVCL_D615) and Expi293S (Thermo Fisher Scientific Cat# A39240) cells were cultured in Expi293 expression medium according to the manufacturer’s instructions and used for expression of ACE2, SARS-CoV-2 spike S-HexaPro, and RBD for the binding assay. FreeStyle 293-F (Thermo Fisher Scientific Cat# R79007, RRID: CVCL_D603) were cultured in FreeStyle 293 expression medium (Thermo Fisher Scientific Cat# 12338002) according to the manufacturer’s instructions and used for expression of SARSr spikes for electron microscopy studies. HEK293T cells (ATCC Cat# CRL-3216, RRID:CVCL_0063) were cultured in DMEM (Thermo Fisher Scientific, Cat# 11960044) supplemented with 10% fetal bovine serum (Omega Scientific, Cat# FB-02), 100U/mL Penicillin/Streptomycin (Corning, Cat# 30-002-CI), and 2mM L-glutamine (Corning, Cat# 25-005-CI) for generating SARS-CoV and SARS-CoV-2 pseudoviruses for the neutralization assay. The cell lines were not further authenticated. (See also METHOD DETAILS).

### Method details

#### Expression and purification of SARS-CoV, SARS-CoV-2 and SARSr-CoV RBDs

The receptor-binding domain (RBD) (residues 319-541) of the SARS-CoV-2 spike (S) protein (GenBank: QHD43416.1), RBD (residues 306-527) of the SARS-CoV S protein (GenBank: ABF65836.1), RBD (residues 315-537) of Guangdong pangolin-CoV (GenBank: QLR06866.1), and RBD (residues 319-541) of Bat-CoV RaTG13 (GenBank: QHR63300.2) were separately cloned into a customized pFastBac vector (Ekiert et al., 2011), and fused with an N-terminal gp67 signal peptide and C-terminal His_6_ tag (Yuan et al., 2020b). Recombinant bacmids encoding each RBDs were generated using the Bac-to-Bac system (Thermo Fisher Scientific) followed by transfection into Sf9 cells using FuGENE HD (Promega) to produce baculoviruses for RBD expression. RBD proteins were expressed in High Five cells (Thermo Fisher Scientific) with suspension culture shaking at 110 rpm at 28°C for 72 h after the baculovirus transduction at an MOI of 5 to 10. Each supernatant containing RBD proteins were then concentrated using a 10 kDa MW cutoff Centramate cassette (Pall Corporation) followed by affinity chromatography using Ni-NTA resin (QIAGEN) and size exclusion chromatography using a HiLoad Superdex 200 pg column (Cytiva). The purified protein samples were buffer exchanged into 20 mM Tris-HCl pH 7.4 and 150 mM NaCl and concentrated for binding analysis and crystallographic studies.

#### Expression and purification of antibodies

Expression plasmids encoding the heavy (HC) and light chains (LC) of the CV38-142 and CV07-250 (Kreye et al., 2020), COVA1-16 and COVA2-39 (Brouwer et al., 2020), CC12.1 (Rogers et al., 2020), and S309 (Pinto et al., 2020) IgG or Fab were transiently co-transfected into ExpiCHO cells at a ratio of 2:1 (HC:LC) using ExpiFectamine CHO Reagent (Thermo Fisher Scientific) according to the manufacturer’s instructions. The supernatant was collected at 14 days post-transfection. The IgG antibodies and Fabs were purified with a CaptureSelect CH1-XL Matrix column (Thermo Fisher Scientific) for affinity purification and a HiLoad Superdex 200 pg column (Cytiva) for size exclusion chromatography. The purified protein samples were buffer exchanged into 20 mM Tris-HCl pH 7.4 and 150 mM NaCl and concentrated for binding analysis, crystallographic studies, negative-stain electron microscopy, and pseudovirus neutralization assays.

#### Expression and purification of human ACE2, SARS-CoV-2 RBD, and S-HexaPro for binding assay

The N-terminal peptidase domain of human ACE2 (residues 19 to 615, GenBank: BAB40370.1) and the receptor-binding domain (RBD) (residues 319-541) of the SARS-CoV-2 spike (S) protein (GenBank: QHD43416.1) were cloned into phCMV3 vector and fused with C-terminal His-tag. A plasmid encoding stabilized SARS-CoV-2 spike protein S-HexaPro (Hsieh et al., 2020) was a gift from Jason McLellan (Addgene plasmid #154754;http://addgene.org/154754; RRID: Addgene_154754) and used to express S-HexaPro for the binding assay. The plasmids were transiently transfected into Expi293F cells using ExpiFectamine 293 Reagent (Thermo Fisher Scientific) according to the manufacturer’s instructions. The supernatant was collected at 7 days post-transfection. The His-tagged ACE2 or S-HexaPro protein were then purified by affinity purification using Ni Sepharose excel resin (Cytiva) followed by size exclusion chromatography.

#### Crystallization and X-ray structure determination

The CV38-142 Fab complexed with SARS-CoV-2 RBD and COVA1-16 Fab (3-mer complex) and CV38-142 Fab complexed with SARS-CoV RBD (2-mer complex) were formed by mixing each of the protein components in an equimolar ratio and incubating overnight at 4°C. 384 conditions of the JCSG Core Suite (QIAGEN) were used for setting-up trays for screening the 3-mer complex (12.1 mg/mL) and 2-mer complex (15.0 mg/mL) on our robotic CrystalMation system (Rigaku) at Scripps Research. Crystallization trials were set-up by the vapor diffusion method in sitting drops containing 0.1 μL of protein complex and 0.1 μL of reservoir solution. Crystals appeared on day 3, were harvested on day 7, pre-equilibrated in cryoprotectant containing 15%–20% ethylene glycol, and then flash cooled and stored in liquid nitrogen until data collection. Diffraction data were collected at cryogenic temperature (100 K) at beamlines 23-ID-D and 23-ID-B of the Advanced Photon Source (APS) at Argonne National Laboratory and processed with HKL2000 (Otwinowski and Minor, 1997). Diffraction data were collected from crystals grown in drops containing 1.0 M lithium chloride, 10% (w/v) polyethylene glycol 6000, 0.1 M citric acid pH 4.0 for the 3-mer complex and drops containing 0.2 M di-ammonium tartrate, 20% (w/v) polyethylene glycol 3350 for the 2-mer complex. The X-ray structures were solved by molecular replacement (MR) using PHASER (McCoy et al., 2007) with MR models for the RBD and Fab from PDB 7JMW (Liu et al., 2020). Iterative model building and refinement were carried out in COOT (Emsley and Cowtan, 2004) and PHENIX (Adams et al., 2010), respectively. Epitope and paratope residues, as well as their interactions, were identified by using PISA program (Krissinel and Henrick, 2007) with buried surface area (BSA > 0 Å^2^) as the criterion.

#### Expression and purification of recombinant spike protein for nsEM

The spike constructs used for negative-stain EM contain the mammalian codon-optimized gene encoding residues 1-1208 (SARS-CoV-2, GenBank: QHD43416.1) and 1-1190 (SARS-CoV, GenBank: AFR58672.1) of the spike protein, followed by a C-terminal T4 fibritin trimerization domain, a HRV3C cleavage site, 8x-His tag and a Twin-Strep tags subcloned into the eukaryotic-expression vector pcDNA3.4. For the SARS-CoV-2 spike protein, three amino-acid mutations were introduced into the S1–S2 cleavage site (RRAR to GSAS) to prevent cleavage and two stabilizing proline mutations (K986P and V987P) to the HR1 domain. Residues T883 and V705 were mutated to cysteines to introduce a disulfide for additional S stabilization. For the SARS-CoV spike protein, residues at 968 and 969 were replaced by prolines to generate stable spike proteins as described previously (Kirchdoerfer et al., 2018). The spike plasmids were transfected into 293F cells and supernatant was harvested at 6 days post transfection. Spike proteins were purified by running the supernatant through streptactin columns and then by size exclusion chromatography using Superose 6 increase 10/300 columns (Cytiva). Protein fractions corresponding to the trimeric spike protein were pooled and concentrated.

#### nsEM sample preparation and data collection

SARS-CoV-2 and SARS-CoV proteins were complexed with six molar excess of Fab for 1 h prior to direct deposition onto carbon-coated 400-mesh copper grids. The EM grids were stained with 2% (w/v) uranyl-formate for 90 s immediately following sample application. Grids were either imaged at 120 keV on a Tecnai T12 Spirit using a 4kx4k Eagle CCD. Micrographs were collected using Leginon (Suloway et al., 2005) and the images were transferred to Appion for processing. Particle stacks were generated in Appion (Lander et al., 2009) with particles picked using a difference-of-Gaussians picker (DoG-picker) (Voss et al., 2009). Particle stacks were then transferred to Relion (Zivanov et al., 2018) for 2D classification followed by 3D classification to sort well-behaved classes. Selected 3D classes were auto-refined on Relion and used to illustrate with UCSF Chimera (Pettersen et al., 2004). A published prefusion spike model (PDB: 6Z97) (Huo et al., 2020) was used in our structural analysis.

#### Measurement of binding affinities and competition using biolayer interferometry

Binding assays were performed by biolayer interferometry (BLI) using an Octet Red instrument (FortéBio). To determine the binding affinity of CV38-142 Fab with SARS-CoV-2 and SARS-CoV RBDs, 20 μg/mL of His-tagged SARS-CoV or SARS-CoV-2 RBD protein purified from Hi5 cell expression was diluted in kinetics buffer (1x PBS, pH 7.4, 0.002% Tween-20, 0.01% BSA) and loaded on Ni-NTA biosensors (ForteBio) for 300 s. After equilibration in kinetics buffer for 60 s, the biosensors were transferred to wells containing serially diluted Fab samples in running buffer to record the real time association response signal. After a 120 s association step, the biosensors were transferred to wells containing blank running buffer to record the real time disassociation response signal. All steps were performed at 1000 rpm shaking speed. K_D_s were determined using ForteBio Octet CFR software. To determine the binding affinity of CV38-142 Fab or S309 IgG with SARS-CoV-2 RBD pretreated with or without PNGase F, Fab or IgG was loaded on Fab2G or AHC biosensors (ForteBio) for 300 s followed by similar steps to test binding to RBD that was expressed in Expi293F cells. For the sandwich binning assay, CV38-142 IgG was loaded onto AHC biosensors (ForteBio) followed by equilibration in kinetics buffer. The biosensors were transferred to wells containing either SARS-CoV-2 RBD or S-HexaPro proteins in kinetics buffer to allow for antigen association for 200 s followed by testing association of a second antibody Fab or ACE2 for 120 s.

#### Measurement of competition using surface plasma resonance

To test whether binding of CV38-142 to SARS-CoV-2 RBD has an impact on the binding of ACE2, a surface plasma resonance (SPR) competition assay was performed on a Biacore T200 instrument (Cytiva) at 25°C. Biotinylated human ACE2 (residue 18-740, ACROBiosystems) was reversibly immobilized on a CAP sensor chip (Cytiva) using Biotin CAPture Kit (Cytiva). CV38-142 IgG used in the SPR assay was produced in CHO cells and was kindly provided by Miltenyi Biotec, Bergisch Gladbach, Germany. The SPR system was primed and equilibrated with running buffer (10 mM HEPES pH 7.4, 150 mM NaCl, 3 mM EDTA, 0.05% Tween 20) before measurement. 10 nM of SARS-CoV-2 RBD (ACROBiosystems) together with different concentrations of CV38-142 IgG dissolved in the running buffer were injected into the system within 90 s in a flow rate of 30 μl/min followed by a regeneration step between each concentration. The binding response signals were recorded in real time by subtracting from reference cell. Experiments were performed in duplicates.

#### Enzyme-linked immunosorbent assay (ELISA) measuring antibody binding to RBD

Rabbit IgG1 Fc-tagged RBD-SD1 regions of MERS-CoV, SARS-CoV and SARS-CoV-2 as well as point mutants thereof (SARS-CoV: N330Q and T332A, SARS-CoV-2: N343Q and T345A) were expressed in HEK293T cells and immobilized onto 96-well plates as previously described (Kreye et al., 2020). Mutations were introduced by overlap extension PCR and confirmed by Sanger sequencing (LGC Genomics). Human anti-spike RBD monoclonal antibodies were applied at 1 μg/mL and detected using horseradish peroxidase (HRP)-conjugated anti-human IgG (Dianova) and the HRP substrate 1-step Ultra TMB (Thermo Fisher Scientific). HRP-conjugated F(ab’)2 anti-rabbit IgG (Dianova) was used to confirm the presence of immobilized antigens.

#### Pseudovirus neutralization assay and synergistic study

Pseudovirus preparation and assay were performed as previously described with minor modifications (Rogers et al., 2020). Pseudovirions were generated by co-transfection of HEK293T cells with plasmids encoding MLV-gag/pol, MLV-CMV-Luciferase, and SARS-CoV-2_Δ18_ spike (GenBank: MN908947) or SARS-CoV spike (GenBank: AFR58672.1). Mutations were introduced by overlapping extension polymerase chain reaction (PCR) to generate mutated spikes of circulating SARS-CoV-2 variants, i.e., B.1.1.7 and B.1.351. The cell culture supernatant containing SARS-CoV-2 and SARS-CoV S-pseudotyped MLV virions was collected at 48 h post transfection and stored at −80°C until use. Lentivirus transduced HeLa cells expressing hACE2 (GenBank: BAB40370.1) were enriched by fluorescence-activated cell sorting (FACS) using biotinylated SARS-CoV-2 RBD conjugated with streptavidin-Alexa Fluor 647 (Thermo, S32357). Monoclonal antibodies IgG or Fab were serially diluted with DMEM medium supplemented with 10% heat-inactivated FBS, 1% Q-max, and 1% P/S. The serial dilutions were incubated with the pseudotyped viruses at 37°C for 1 h in 96-well half-well plate (Corning, 3688). After the incubation, 10,000 HeLa-hACE2 cells were added to the mixture and supplemented 20 μg/mL Dextran (Sigma, 93556-1G) for enhanced infectivity. The supernatant was removed 48 h post incubation, and the cells were washed and lysed in luciferase lysis buffer (25 mM Gly-Gly pH 7.8, 15 mM MgSO_4_, 4 mM EGTA, 1% V/V Triton X-100). After addition of Bright-Glo (Promega, PR-E2620) according to the manufacturer’s instruction, luminescence signal was measured in duplicate. At least two biological replicates were performed for neutralization assays with SARS-CoV-2, circulating SARS-CoV-2 variants of concern, and SARS-CoV. The IgG half-maximal inhibitory concentration (IC_50_) values were calculated using “One Site - Fit LogIC50” regression in GraphPad Prism 9. For synergy assessment of two monoclonal antibodies, an antibody cocktail matrix was prepared by a combination of mixing a fixed concentration of CV38-142 and increasing the concentration of COVA1-16 or increasing the concentration of CV38-142 and fixing the concentration of COVA1-16. Neutralization percentages for each combination were measured and calculated the same way as the pseudovirus neutralization assay. The neutralization data were converted to the input format for the synergy program (Wooten and Albert, 2020). Synergy scores were calculated by fitting the multidimensional synergy of combinations (MuSyC) model, which is a synergy model based on a multidimensional extension of the Hill equation that allows non-linear dose-response surface contour (Meyer et al., 2019). MuSyC model quantifies synergy in bidirectional way and distinguishes synergies between potency and efficacy. The synergy parameter α_12_, namely synergistic potency quantifies how the second antibody changes the potency of the first and α_21_ quantifies how the first antibody changes the potency of the second. The MuSyC model fitting with the synergy program also gives two other parameters, namely synergistic efficacy (β) and synergistic cooperativity (γ) score (Wooten and Albert, 2020). The β score denotes synergistic efficacy, which quantifies the percent change on the maximal efficacy of the antibody combination compared to the most efficacious single agent. The γ_12_ score denotes how the first antibody changes the second Hill slope, while γ_21_ denotes how the second changes the first Hill slope.

#### Shape complementarity analysis

Shape complementarity values (Sc) were calculated using SC program as described previously (Lawrence and Colman, 1993).

### Quantification and statistical analysis

The kinetics binding data were analyzed using Octet Data Analysis software version 12.0 (Sartorius). Pseudovirus neutralization data from at least two biological repeats were analyzed in Prism 9 (GraphPad) using “One Site - Fit LogIC50” regression model. Data are shown as mean ± SD 2D matrix neutralization data were analyzed using synergy program (Wooten and Albert, 2020) with the multidimensional synergy of combinations (MuSyC) model (Meyer et al., 2019). (see also figure legends and METHOD DETAILS).

## Supplemental information

Document S1. Figure S1–S6 and Tables S1 and S2

Document S2. Article plus supplemental information
