# Supplementary material for: A combination of cross-neutralizing antibodies synergizes to prevent SARS-CoV-2 and SARS-CoV pseudovirus infection
Source: Cell Host Microbe. 2021 May 12;29(5):806–818.e6. doi: 10.1016/j.chom.2021.04.005 (PMC8049401; doi:10.1016/j.chom.2021.04.005)
Supplement: Document S1. Figure S1–S6 and Tables S1 and S2 [file mmc1.pdf]

Cell Host & Microbe, Volume *29*

Supplemental information

A combination of cross-neutralizing antibodies synergizes to prevent SARS-CoV-2 and SARS-CoV pseudovirus infection

Hejun Liu, Meng Yuan, Deli Huang, Sandhya Bangaru, Fangzhu Zhao, Chang-Chun D. Lee, Linghang Peng, Shawn Barman, Xueyong Zhu, David Nemazee, Dennis R. Burton, Marit J. van Gils, Rogier W. Sanders, Hans-Christian Kornau, S. Momsen Reincke, Harald Prüss, Jakob Kreye, Nicholas C. Wu, Andrew B. Ward, and Ian A. Wilson

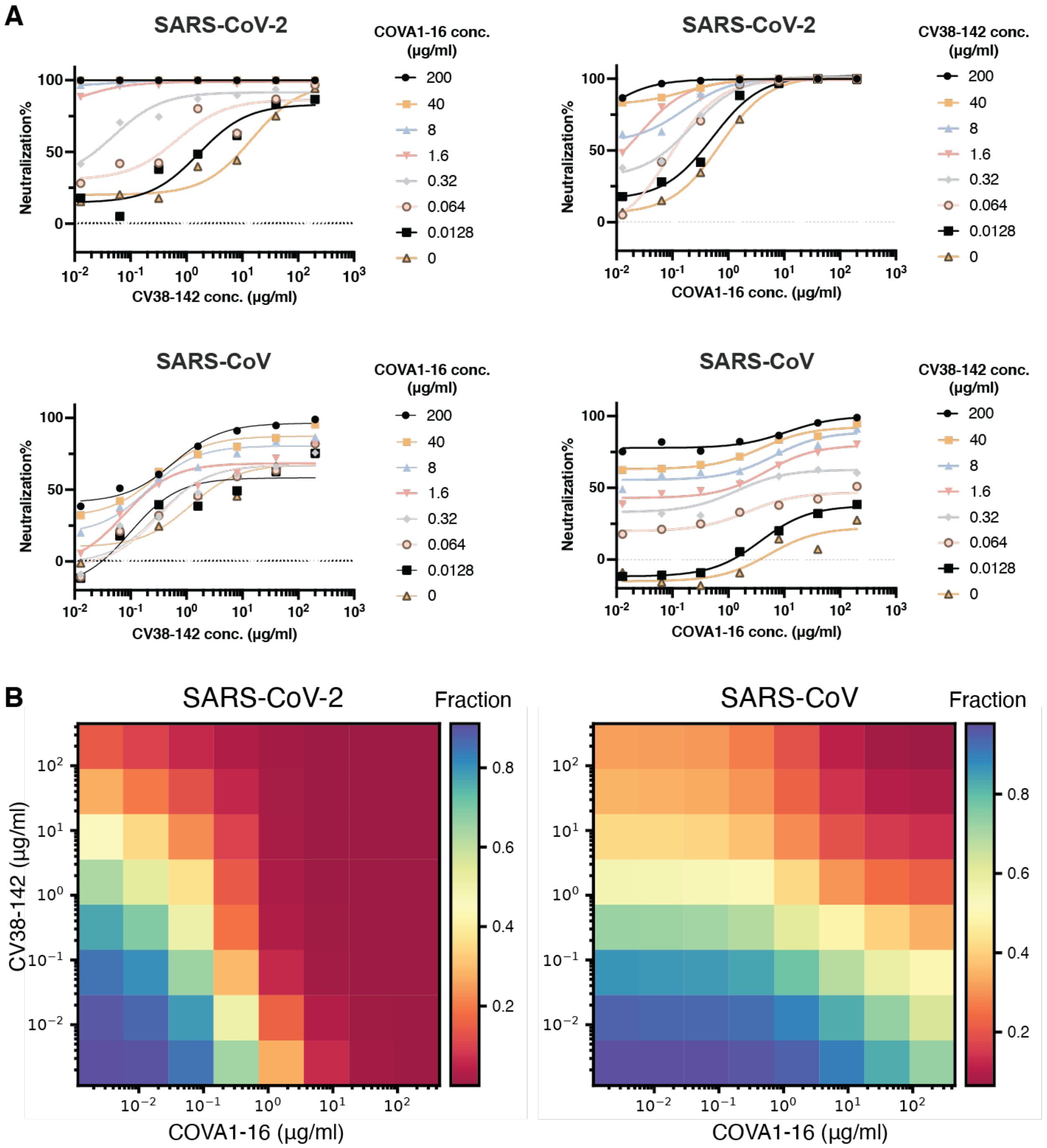

Figure S1, related to Figure 2. Neutralization curves and synergy quantification. A. Neutralization curves for the 2D neutralization matrix assay. The four panels show the actual neutralization curves from 2D neuralization matrix assay using mixtures of one antibody with a fixed concentration (curve legend) and the other with increasing concentration (x-axis). For SARS-CoV-2 neutralization assay, increasing the concentration of COVA1-16 substantially increased

the potency (i.e. half-maximal neutralization concentration) of CV38-142 (upper left panel) and vice versa (upper right panel). For the SARS-CoV neutralization assay, increasing the concentration of COVA1-16 substantially increased the potency (i.e. half-maximal neutralization concentration) of CV38-142 (lower left panel) and vice versa (lower right panel). Increasing the concentration of COVA1-16 also substantially increased the neutralization efficacy of CV38-142 (i.e. maximum percentage of neutralization) (lower left panel). **B.** MuSyC model analysis. Neutralization percentage was used to generate the fraction data with 1 indicates no neutralization and 0 indicates 100% neutralization. Heatmap plot shows the fraction data used for synergy quantification. $A>1$, $\gamma>1$, or $\beta>0$ indicate synergism while $\alpha<1$, $\gamma<1$, or $\beta<0$ indicate antagonism. CV38-142 was assigned as the first antibody and COVA1-16 was assigned as the second antibody in the analysis. For SARS-CoV-2, CV38-142 and COVA1-16 synergistically change each other's neutralization potency (α_{21}=5314, α_{12}=671) and CV38-142 increase the steepness of COVA1-16's neutralization Hill slope, while COVA1-16 decrease the steepness of CV38-142's neutralization Hill slope (γ_{21}= 2.1, γ_{12}=0.38). For SARS-CoV, CV38-142 and COVA1-16 synergistically change each other's neutralization potency (α_{21}=27, α_{12}=123) and COVA1-16 increased the efficacy of CV38-142 as indicated by the positive synergistic efficacy score (β=0.4). However, the synergistic efficacy (β) in SARS-CoV-2 neutralization and synergistic cooperativity (γ) in SARS-CoV neutralization are ambiguous (not interpretable) at a 95% confidence interval.

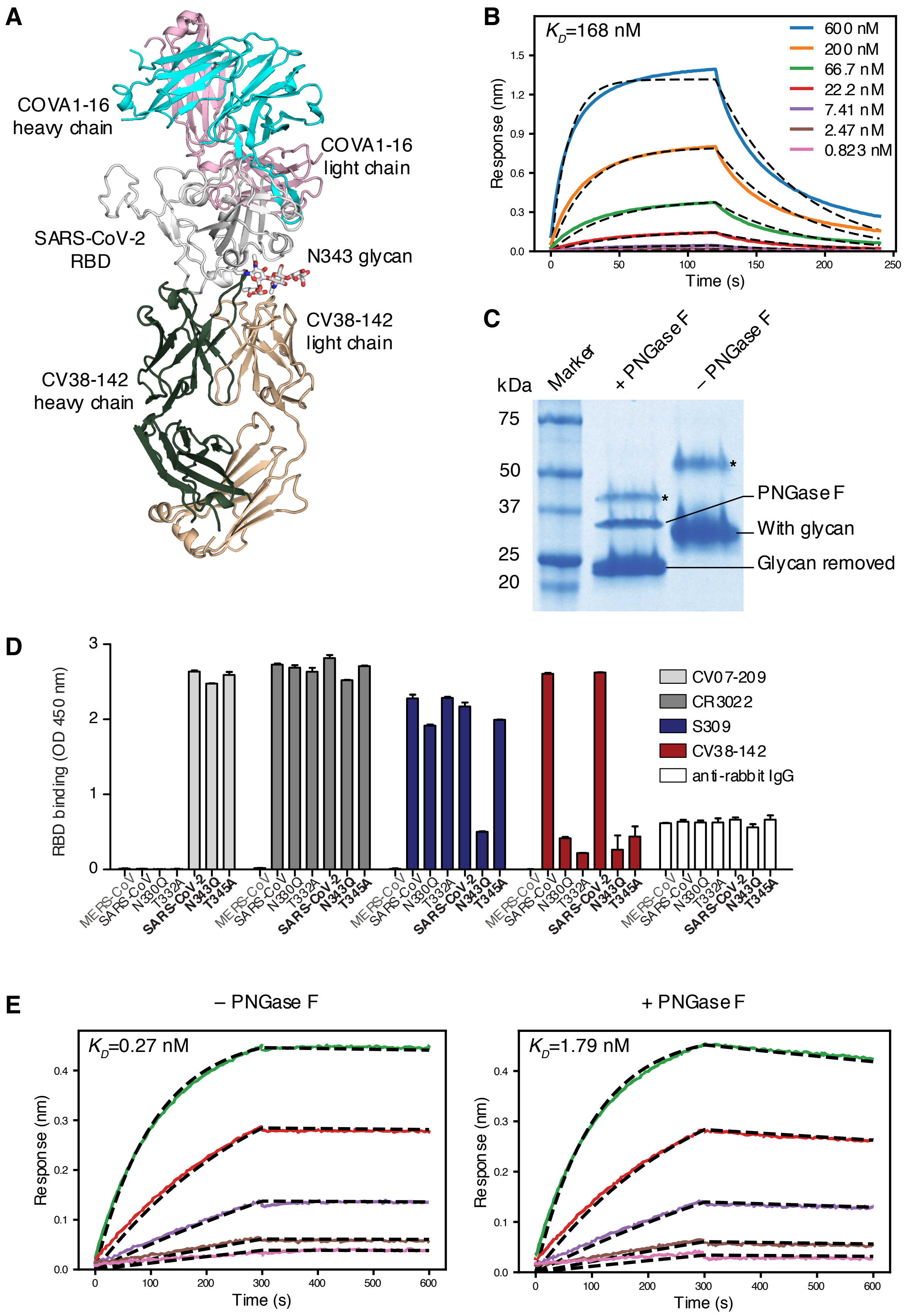
A
COVA1-16 heavy chain
COVA1-16 light chain
SARS-CoV-2 RBD
N343 glycan
CV38-142 light chain
CV38-142 heavy chain
B
K_D=168 nM
600 nM
200 nM
66.7 nM
22.2 nM
7.41 nM
2.47 nM
0.823 nM
Response (nm)
Time (s)
C
kDa
Marker
+ PNGase F
– PNGase F
75
50
37
25
20
PNGase F
With glycan
Glycan removed
D
RBD binding (OD 450 nm)
CV07-209
CR3022
S309
CV38-142
anti-rabbit IgG
MERS-CoV
SARS-CoV
N330Q
T332A
SARS-CoV-2
N343Q
T345A
E
– PNGase F
+ PNGase F
K_D=0.27 nM
K_D=1.79 nM
Response (nm)
Time (s)

Figure S2, related to Figure 3. The N343 glycan in the RBD is involved in binding to CV38-142. A. Crystal structures of SARS-CoV-2 RBD in complex with CV38-142 and COVA1-16 Fabs. Ribbon representation of SARS-CoV-2 complexed with both CV38-142 Fab and COVA1-16 Fab. The N343 glycan is shown in sticks. SARS-CoV-2 RBD is in white, CV38-142 heavy chain in forest green and light chain in wheat, and COVA1-16 heavy chain in cyan and light chain in pink. There is no overlap between COVA1-16 and CV38-142 epitope as well as no interaction between COVA1-16 Fab and CV38-142 Fab when bound to the same RBD. **B.** Decreased binding affinity between CV38-142 Fab and SARS-CoV-2 RBD expressed in HEK293S cell. HEK293S cell does not have N-acetylglucosaminyltransferase I (GnTI) and, therefore, protein expressed in this cell lack complex N-glycans (Reeves et al., 2002). N343 glycan of SARS-CoV-2 RBD expressed in HEK293S cell has no fucose moiety and abolishes its interaction to CV38-142 as shown in Figure 3B. Binding kinetics were measured by biolayer interferometry (BLI) with RBDs on the biosensor and Fab in solution. Concentrations of Fab serial dilution are shown in upper right insert. The association and disassociation were recorded in real time (s) on the x axis with binding response (nm) on the y axis with colored lines. Disassociation constant (K_D) values were obtained by fitting a 1:1 binding model. The fitted curves are represented by the dash lines (black). **C.** PNGase F treatment removes glycans in the SARS-CoV-2 RBD. Non-reducing sodium dodecyl sulphate–polyacrylamide gel electrophoresis (SDS-PAGE) showed the shifted bands between treated and untreated SARS-CoV-2 RBD. Lanes of protein marker, PNGase F treated sample, control sample are indicated above the gel. Protein bands corresponding to SARS-CoV-2 RBD with glycan, with glycan removal, and PNGase F are labeled. Asterisk (*) indicates a small fraction of dimeric RBD formed during protein production. **D.** Mutation in the N343 sequon results in a large decrease in CV38-142 binding to the SARS-CoV-2 RBD**.** Rabbit IgG1 Fc-tagged RBDs of MERS-CoV, SARS-CoV, SARS-CoV-2 as well as mutant RBDs were coated on a 96-well plate. Binding of the indicated anti-RBD antibodies was tested using an enzyme-linked immunosorbent

assay (ELISA). Abolishing the N343 glycosylation by introducing either N343Q or T345A in SARS-CoV-2 and N330Q or T332A in SARS-CoV RBD substantially decreased the CV38-142 binding, with no obvious loss on binding by other antibodies such as CR3022 and CV07-209. S309 appears to be less susceptible to the absence of N343 glycosylation. Note that S309 binds T345A stronger than N343Q, although both mutations lead to no glycosylation at residue 343. N343Q in SARS-CoV-2 may either lead to some steric clashes for antibody binding to the N343Q site that is not the case for T345A or these mutations might result in a less stable RBD that interferes with the binding detection, as seen by deep mutational scanning (Starr et al., 2020). Two independent repeats were performed, and bar values indicate mean RBD binding with error bars represent the standard deviation. **E.** N343 glycan aids S309 binding to SARS-CoV-2 RBD (Pinto et al., 2020). The binding kinetics were measured by BLI with S309 on the biosensor and RBD in solution. SARS-CoV-2 RBD was treated with or without PNGase F digestion in the same concentration and condition before being used in the BLI assay. Concentrations of RBD serial dilution are color coded as in **B**. The association and disassociation were recorded in real time (s) on the x axis and response (nm) on the y axis as colored lines. Disassociation constant (K_D) values were obtained by fitting a 1:1 binding model with fitted curves represented by the dash lines.

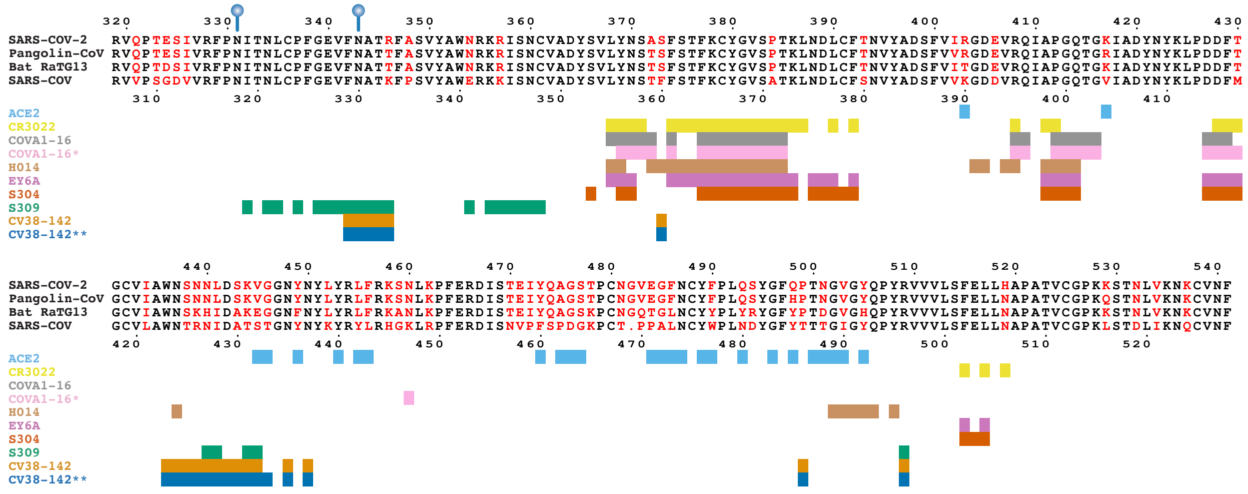

Figure S3, related to Figure 4. CV38-142 epitope and comparison with other cross-reactive antibodies. Epitopes of cross-reactive antibodies on SARS-CoV-2 or SARS-CoV RBD. Sequence alignment of CV38-142 reactive RBDs from SARS-CoV-2, SARS-CoV, bat coronavirus RaTG13, and Guangdong pangolin coronavirus RBD with non-conserved residues highlighted in red. The conserved glycosylation sites are marked with blue balloons. Numbers corresponding to SARS-CoV-2 RBD and SARS-CoV RBD are labelled every ten residues above and below the sequences panel. Colored bars representing the RBD epitope residues corresponding to each antibody or ACE2 are shown under the sequence panel with their ligand name (ACE2 or antibody) on the left. Epitope residues or ACE2-interacting residues are assigned as BSA>0 Å^2 as calculated by the PISA program (Krissinel and Henrick, 2007) for SARS-CoV-2 RBD with ACE2 (PDB: 6M0J), CR3022(PDB: 6XC3), COVA1-16(PDB: 7JMW), H014(PDB: 7CAH), EY6A(PDB: 6ZCZ), S304 and S309 (PDB: 7JX3). * indicates COVA1-16 epitope on SARS-CoV-2 RBD calculated from its structure complexed with CV38-142 Fab and SARS-CoV-2 RBD reported in this study as compared to that without CV38-142 (above). The slight discrepancy in COVA1-16 epitope residues is due to the improvement in resolution rather than the simultaneous binding of

CV38-142. ** indicates CV-38-142 epitope on SARS-CoV RBD reported in this study in comparison to that on SARS-CoV-2 RBD also reported in this study (above).

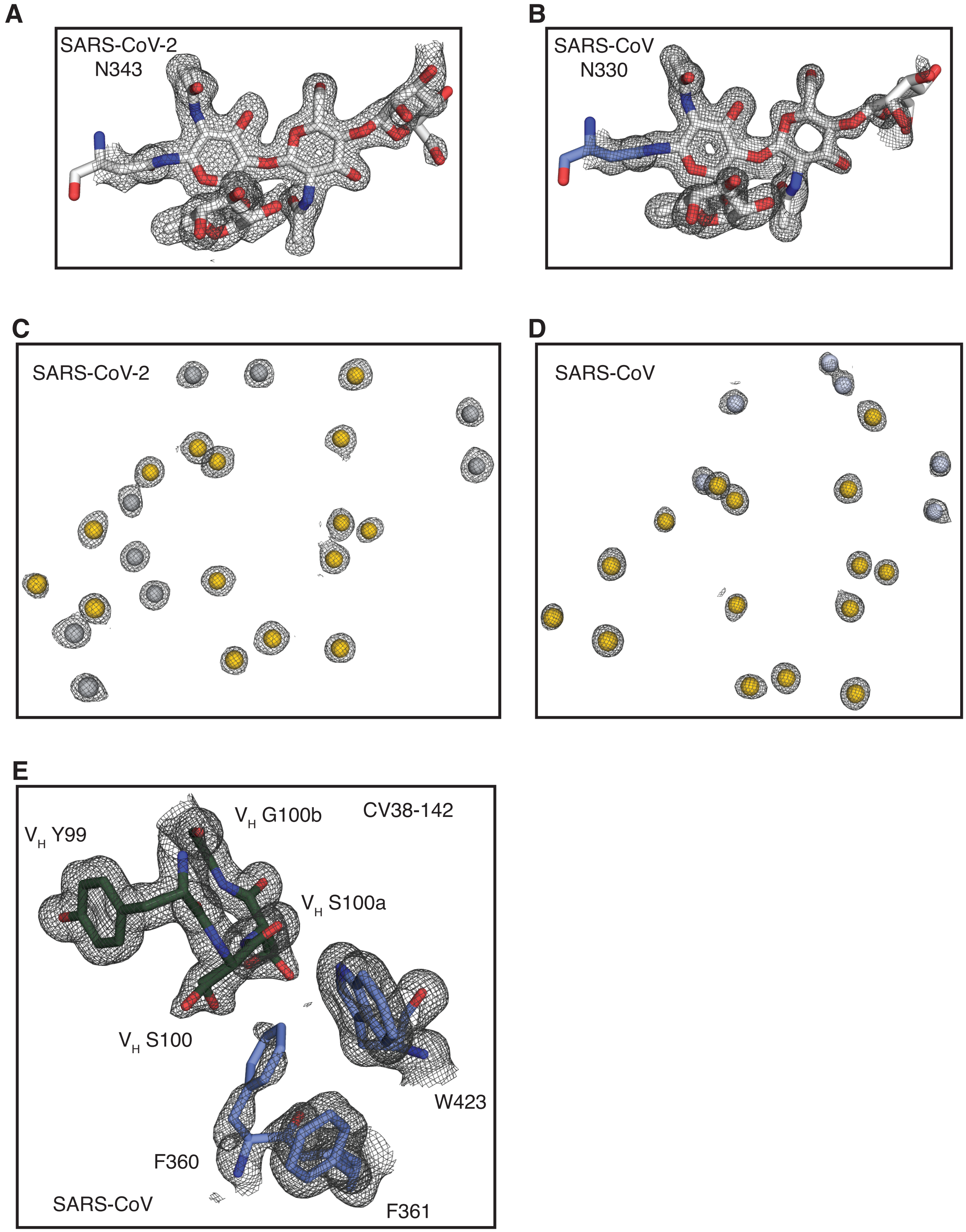

A
SARS-CoV-2
N343
B
SARS-CoV
N330
C
SARS-CoV-2
D
SARS-CoV
E
V H G100b
CV38-142
V H Y99
V H S100a
V H S100
W423
F360
SARS-CoV
F361

Figure S4, related to Figures 3 and 5. Electron density for glycans, water molecules and region around F360 in the RBD. 2mFo-DFc Sigma-A weighted maps were calculated by Phenix software and contoured at 1.0 σ to show electron density in mesh with the refined structure in spheres (water molecules) or sticks. Glycans and residues are shown in sticks. Water molecules are shown in spheres. Maps are shown in grey meshes. **A.** Electron density for the SARS-CoV-2 N343 glycan. **B.** Electron density for the SARS-CoV N330 glycan. **C.** Electron density for waters in the interface between CV38-142 and SARS-CoV-2 RBD. Shared waters interacting to both SARS-CoV-2 and SARS-CoV RBD are highlighted in yellow. **D.** Electron density for waters in the interface between CV38-142 and SARS-CoV RBD. **E.** Electron density for F360 of SARS-CoV RBD and its surrounding residues. CV38-142 is in forest green and SARS-CoV RBD in pale blue.

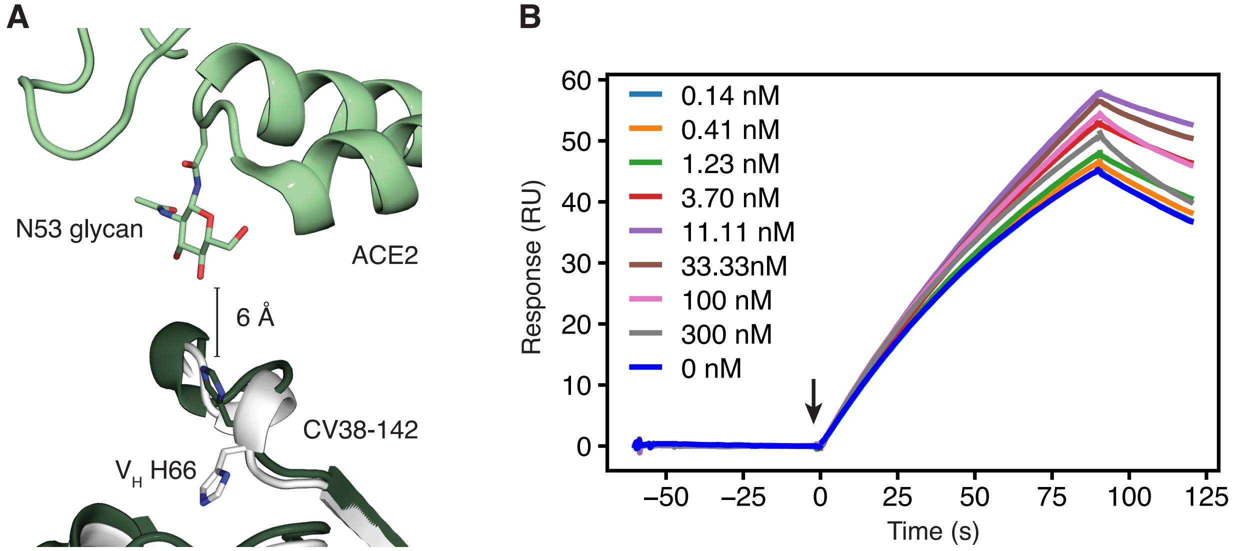

Figure S5, related to Figure 4. A. Close-up view of S60-H66 region of the CV38-142 Fab that is close to the N53 glycan of ACE2 if both were to bind SARS-CoV-2 RBD. Structures of CV38-142 Fab + SARS-CoV-2 RBD (forest green), CV38-142 Fab + SARS-CoV RBD (white), and ACE2 + SARS-CoV-2 spike are aligned by superimposition of their RBD. V_H H66 of CV38-142 in the Fab complex with SARS-CoV-2 and SARS-CoV RBD is shown as sticks. The closest distance between ACE2 and CV38-142 is 6 Å, while V_H H66 as well as the rest of region S60-H66 of CV38-142 shows some flexibility to accommodate the N53 glycan of ACE2. **B.** Surface plasma resonance (SPR) competition assay. Human ACE2 was immobilized on a CAP sensor chip before the measurement of competition. Binding to ACE2 was monitored in real time. Arrow indicates the timepoint of injection of SARS-CoV-2 RBD + CV38-142 IgG mixture. The concentration of SARS-CoV-2 RBD was fixed at 10 nM while the concentration of CV38-142 IgG was increased as indicated in the insert legend. No inhibition from CV38-142 IgG was observed as all concentrations tested give very similar on- and off-rates for the binding of ACE2 to the given concentration of SARS-CoV-2 RBD.

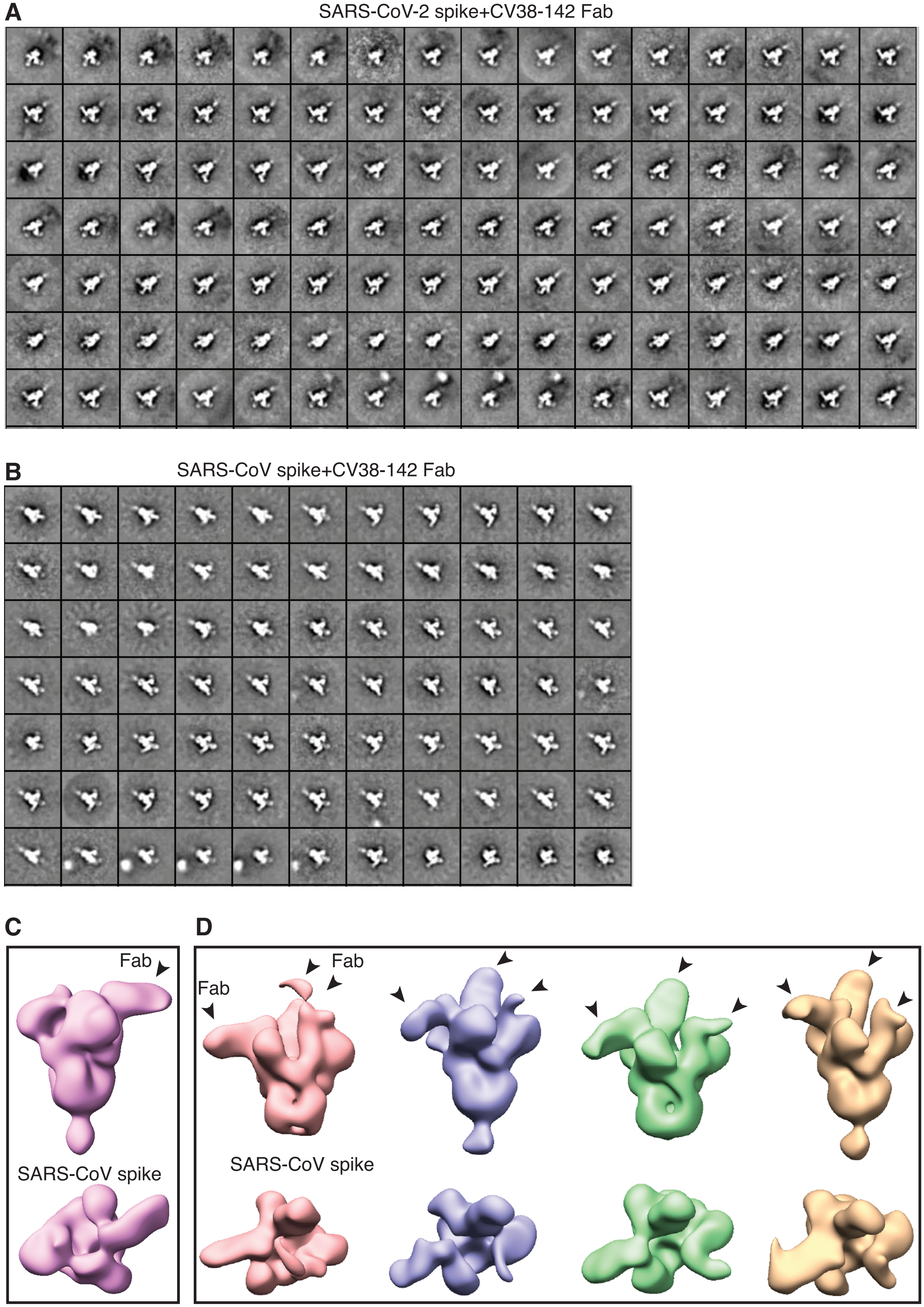
A
SARS-CoV-2 spike+CV38-142 Fab
B
SARS-CoV spike+CV38-142 Fab
C
Fab
SARS-CoV spike
D
Fab
Fab
SARS-CoV spike

Figure S6, related to Figure 6. nsEM 2D images and 3D reconstruction of CV38-142 Fab in complex with SARS-CoV-2 and SARS-CoV spikes. A-B. 2D classification of the nsEM images showing various binding stoichiometries between CV38-142 Fab and SARS-CoV-2 spike (**A**) and SARS-CoV spike (**B**). **C-D.** 3D reconstruction of SARS-CoV spike bound with one CV38-142 Fab (**C**) and three CV38-142 Fabs (**D**). Arrow heads indicate the RBD with Fab bound. **C.** The spike with at least one "up" RBD. CV38-142 Fab binds to the RBD in the "up" conformation. **D.** The spike with at least one "up" RBD and one "down" RBD. Fabs show binding at various angles among these 3D reconstructions due to flexibility of the RBD in the spike and whether the RBD is up or down. The EM maps for some RBDs bound to Fab are difficult to interpret due to the heterogeneous conformations resulting from the RBD flexibility.

Table S1, related to Figure 3. Crystallographic data collection and refinement statistics

	CV38-142 Fab + SARS-CoV-2 RBD + COVA1-16 Fab	CV38-142 Fab + SARS-CoV RBD
**Data collection**		
Beamline	APS 23ID-D	APS 23ID-B
Wavelength (Å)	0.97934	1.03317
Space group	$P\,2_1\,2_1\,2_1$	C 1 2 1
Unit cell parameters		
a, b, c (Å)	59.7, 148.2, 162.3	238.0, 71.9, 49.2
α, β, γ (°)	90, 90, 90	90, 90.8, 90
Resolution (Å) [a]	50.0–1.94 (1.97–1.94)	50.0 –1.53 (1.56–1.53)
Unique reflections [a]	107,834 (5,287)	120,261 (5,241)
Redundancy [a]	10.2 (8.3)	6.4 (3.8)
Completeness (%) [a]	100 (100)	95.7 (84.1)
$<I/\sigma_I>$ [a]	29.2 (2.5)	24.5 (1.1)
R_{sym}[b] (%) [a]	8.6 (87.3)	9.1 (79.4)
R_{pim}[b] (%) [a]	2.8 (32.2)	3.9 (41.9)
$CC_{1/2}$[c] (%) [a]	99.8 (73.4)	99.1 (64.7)
**Refinement statistics**		
Resolution (Å)	33.7–1.94	49.2–1.53
Reflections (work)	101,477	114,184
Reflections (test)	5,355	6,011
R_{cryst}[d] / R_{free}[e] (%)	16.9/20.0	17.0/19.2
No. of atoms	9,315	5619
Macromolecules	8,195	4912
Glycans	49	74
Solvent	1,071	633
Average B-value (Å^2)	23	27
Macromolecules	22	26
Fab	22	24
RBD	23	30
Glycan	32	32
Solvent	32	37
Wilson B-value (Å^2)	19	19
**RMSD from ideal geometry**		
Bond length (Å)	0.011	0.011
Bond angle (°)	1.07	1.45
**Ramachandran statistics (%)**		
Favored	97.5	97.4
Outliers	0.0	0.0
**PDB code**	7LM8	7LM9

[a] Numbers in parentheses refer to the highest resolution shell.

[b] $R_{sym} = \Sigma_{hkl}\, \Sigma_i \mid I_{hkl,i} - <I_{hkl}> \mid / \Sigma_{hkl}\, \Sigma_i\, I_{hkl,i}$ and $R_{pim} = \Sigma_{hkl}\, (1/(n-1))^{1/2}\, \Sigma_i \mid I_{hkl,i} - <I_{hkl}> \mid / \Sigma_{hkl}\, \Sigma_i\, I_{hkl,i}$, where $I_{hkl,i}$ is the scaled intensity of the ith measurement of reflection h, k, l, $<I_{hkl}>$ is the average intensity for that reflection, and n is the redundancy.

[c] $CC_{1/2}$ = Pearson correlation coefficient between two random half datasets.

[d] $R_{cryst} = \Sigma_{hkl} \mid F_o - F_c \mid / \Sigma_{hkl} \mid F_o \mid \times 100$, where F_o and F_c are the observed and calculated structure factors, respectively.

[e] R_{free} was calculated as for R_{cryst}, but on a test set comprising 5% of the data excluded from refinement.

Table S2, related to Figure 4. Polar interactions identified at the antibody-antigen interface using the PISA program*

Chain	Residue	Atom	Distance (Å)	Chain	Residue	Atom
**SARS-CoV-2**						
M	SER 100	N	2.9	A	ASN 343	OD1
M	ARG 58	NH1	2.8	A	LEU 441	O
M	SER 55	O	3.2	A	ASN 450	ND2
M	ASP 56	OD2	3.7	A	ASN 450	ND2
M	THR 57	O	2.9	A	LYS 444	NZ
N	TYR 92	O	3.6	A	ASN 440	ND2
M	ASP 56	OD1	2.9	A	LYS 444	NZ
**SARS-CoV**						
H	SER 100	N	3.2	A	ASN 330	OD1
H	ARG 58	NH1	2.7	A	ILE 428	O
H	SER 55	O	3.1	A	ASN 437	ND2
L	TYR 92	O	3.2	A	ASN 427	ND2

*Direct polar interactions of CV38-142 with SARS-CoV-2 RBD that are lost on binding to SARS-CoV are highlighted in yellow.
